# Supplementary material for: KDM6B promotes activation of the oncogenic CDK4/6-pRB-E2F pathway by maintaining enhancer activity in MYCN-amplified neuroblastoma
Source: Nat Commun. 2021 Dec 10;12:7204. doi: 10.1038/s41467-021-27502-2 (PMC8664842; doi:10.1038/s41467-021-27502-2)
Supplement: Supplementary file 1 — Supplementary Information [file 41467_2021_27502_MOESM1_ESM.pdf]

Supplementary Information

a

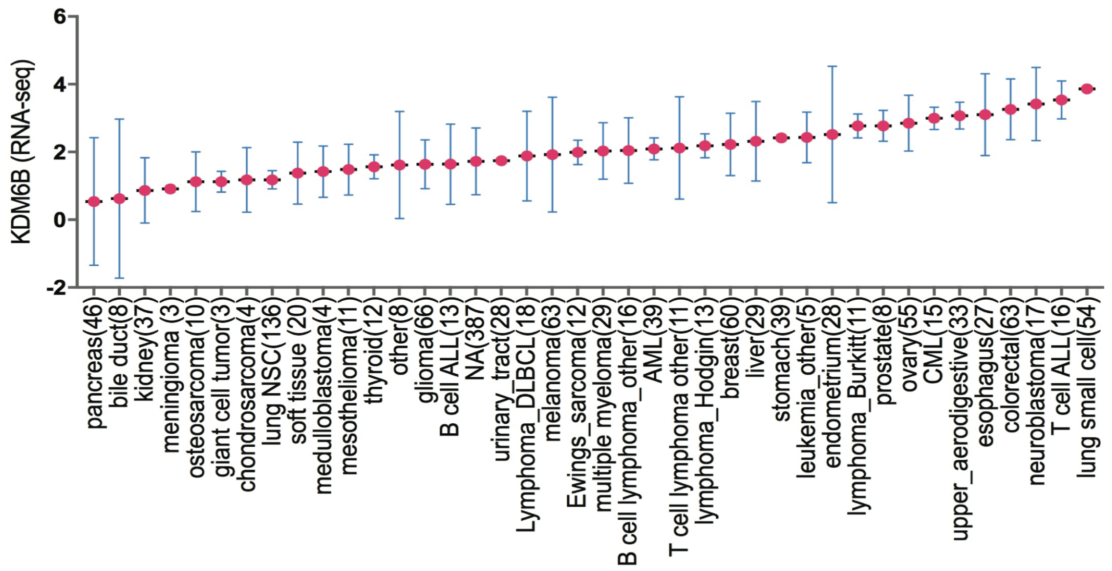

b

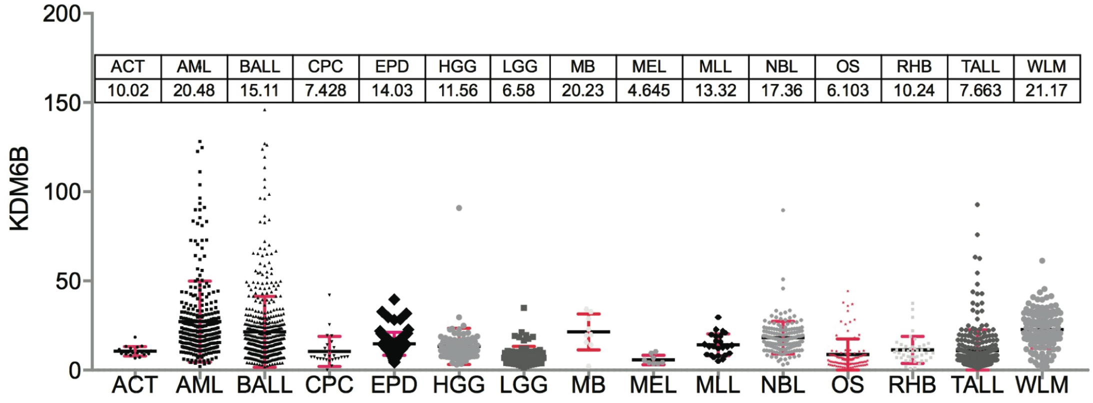

Supplementary Fig. 1 KDM6B is highly expressed in human neuroblastoma. a The expression of *KDM6B* from different cancer lineages. The RNA-seq data was downloaded from https://portals.broadinstitute.org/ccle. Y-axis represents the Fragment Per Kilobase of transcript per Million (FPKM) mapped reads. Sample size for each type of cancer lineage is indicated in bracket of x-axis. Data are represented as mean ± SD. **b** The expression of *KDM6B* from different cancer lineages. The RNA-seq data was downloaded from https://pecan.stjude.cloud. Y-axis represents the Fragment Per Kilobase of transcript per Million (FPKM) mapped reads. Each dot represents one tumor sample. X-axis indicates the tumor types. N=20 for ACT (Adrenocortical tumor), n=306 for AML (Acute myeloid leukemia), n=531 for BALL(B-cell acute lymphoblastic leukemia), n=27 for CPC (Choroid plexus carcinoma), n=82 for HGG (High-grade glioma), n=78 for LGG (Low-grade glioma), n=9 for MB (Medulloblastoma), n=8 for MEL (Merkel cell carcinoma), n=24 for MLL (Mixed-lineage leukemia),

n=160 for NBL (Neuroblastoma), n=107 for OS (Osteosarcoma), n=45 for RHB (Rhabdomyosarcoma), n=264 for TALL (T-cell acute lymphoblastic leukemia), n=125 for WML (Wilm's tumor). Data are represented as mean ± SD.

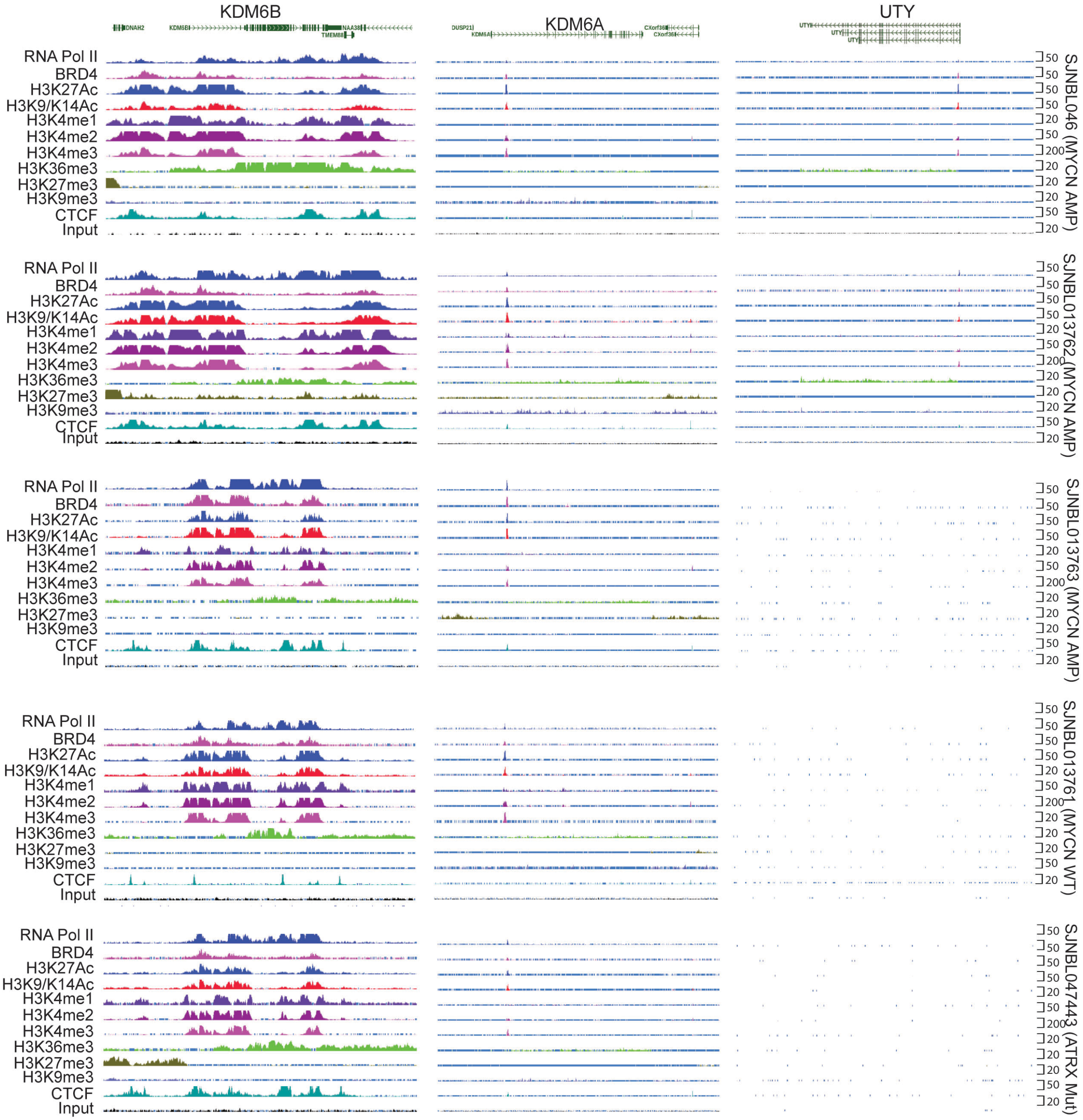

Supplementary Fig. 2 Epigenetic landscape of KDM6A, KDM6B and UTY. The epigenetic landscapes consisting of histone marks and transcription factor binding distinguish KDM6B from KDM6A and UTY in primary neuroblastoma tissues with *MYCN* amplification (SJNBL046, SKNBL013762, SJNBL013763), without *MYCN* amplification (SJNBL013761), or with *ATRX* mutation (SJNBL047443).

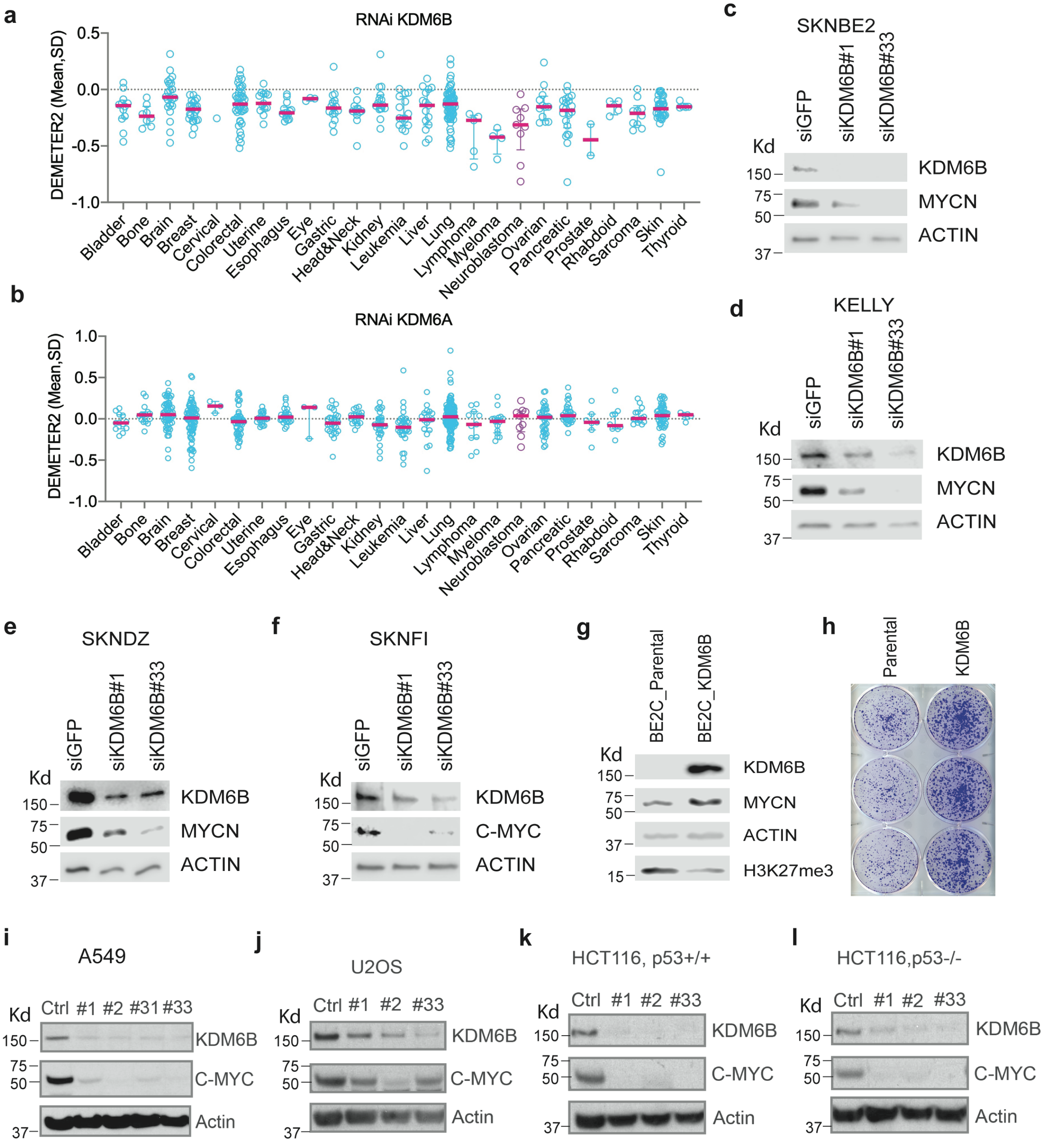

Supplementary Fig. 3 KDM6B regulates MYC expression. a, b The DEMETER2 score of an RNAi screen across 25 cancer lineages. DEMETER2 is a model that scores gene dependencies in 712 cancer cell lines that have been evaluated in three different large-scale pooled RNAi screens (DepMap.org). The y-axis represents DEMETER2 score. The x-axis indicates cancer lineages. Each dot represents one cancer cell line. N= 15 for bone, n=55 for brain, n=77 for breast, n=3 for cervical, n=46 for colorectal, n=20 for uterine, n=24 for esophagus, n=3 for eye, n= 26 for gastric, n=19 for head and neck, n=28 for kidney,

n=34 for leukemia, n=18 for liver, n=134 for lung, n=11 for lymphoma, n=16 for myeloma, n=9 for neuroblastoma, n=37 for ovarian, n=32 pancreatic, n=7 for prostate, n=10 for rhabdoid, n=14 for sarcoma, n=46 for skin, n=5 for thyroid. The red bar represents the mean± SD value of DEMETER2 score. **c-f** Western blot analysis with indicated antibodies to assess MYCN or C-MYC expression after 3-day transfection of 2 different siRNAs to knockdown KDM6B in neuroblastoma cell lines. **g** Western blot analysis with indicated antibodies after BE2C cells are transduced with MSCV-KDM6B. **h** BE2C cells and KDM6B overexpressing cells were seeded at equal numbers in a 6-well plate. 5 days later, cells were stained with crystal violet. **i-l** Western blot analysis with indicated antibodies to assess MYCN or C-MYC expression after 3-day transfection of 2 different siRNA to knockdown KDM6B in lung adenocarcinoma A549 (**i**), osteosarcoma U2OS (**j**), colorectal HCT116 (**k**) and p53 null isogenic HCT116 cells (**l**). The blots are representative of three independent experiments in **c**, **d**, **e-g**, **i-l**.

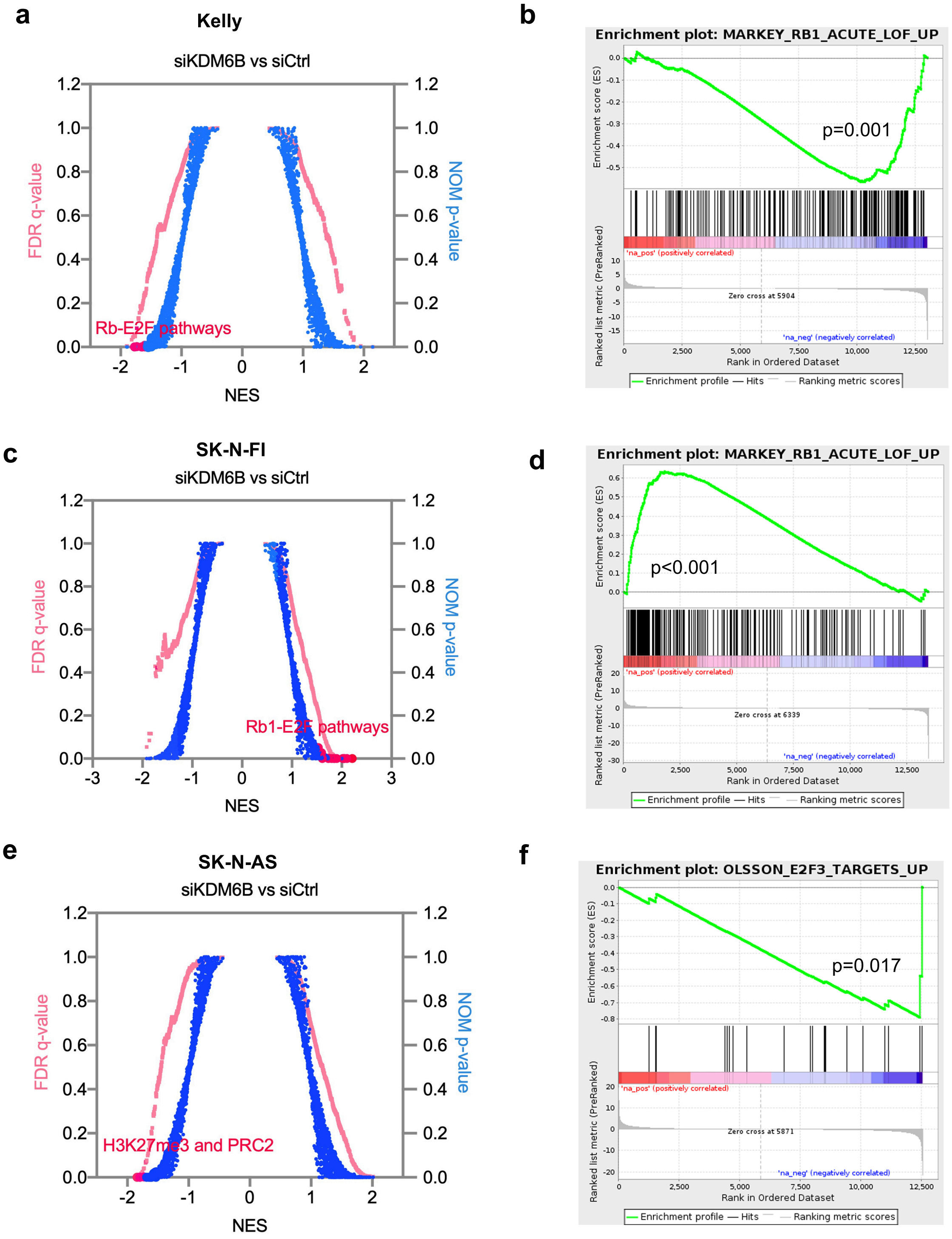

Supplementary Fig. 4 GSEA analyses for KELLY, SK-N-FI and SK-N-AS cells. (**a, c, e**) Quantitative comparison of all chemical and genetic perturbation gene sets from the MSigDB by gene set enrichment analysis (GSEA) for reduced (left) and increased (right) expression of global genes caused by KDM6B knockdown in KELLY (**a**), SK-N-FI (**c**) and SK-N-AS (**e**) cell lines. Data are presented as a scatterplot of normalized p value (right y-axis)/false discovery q value (left y-axis) vs normalized enrichment score

(NES))(x-axis) for each evaluated gene set. The gene sets circled in red color indicate cell cycle, pRB-E2F and MYC pathway gene sets. P value calculated by one-sided Fisher's exact test. The FDR is calculated by comparing the distribution of normalized enrichment scores from many different genesets. (**b, d, f**) Representative GSEA samples for Kelly (**b**), SK-N-FI (**d**) and SK-N-AS (**f**). P value calculated by one-sided Fisher's exact test. The FDR is calculated by comparing the distribution of normalized enrichment scores from many different genesets.

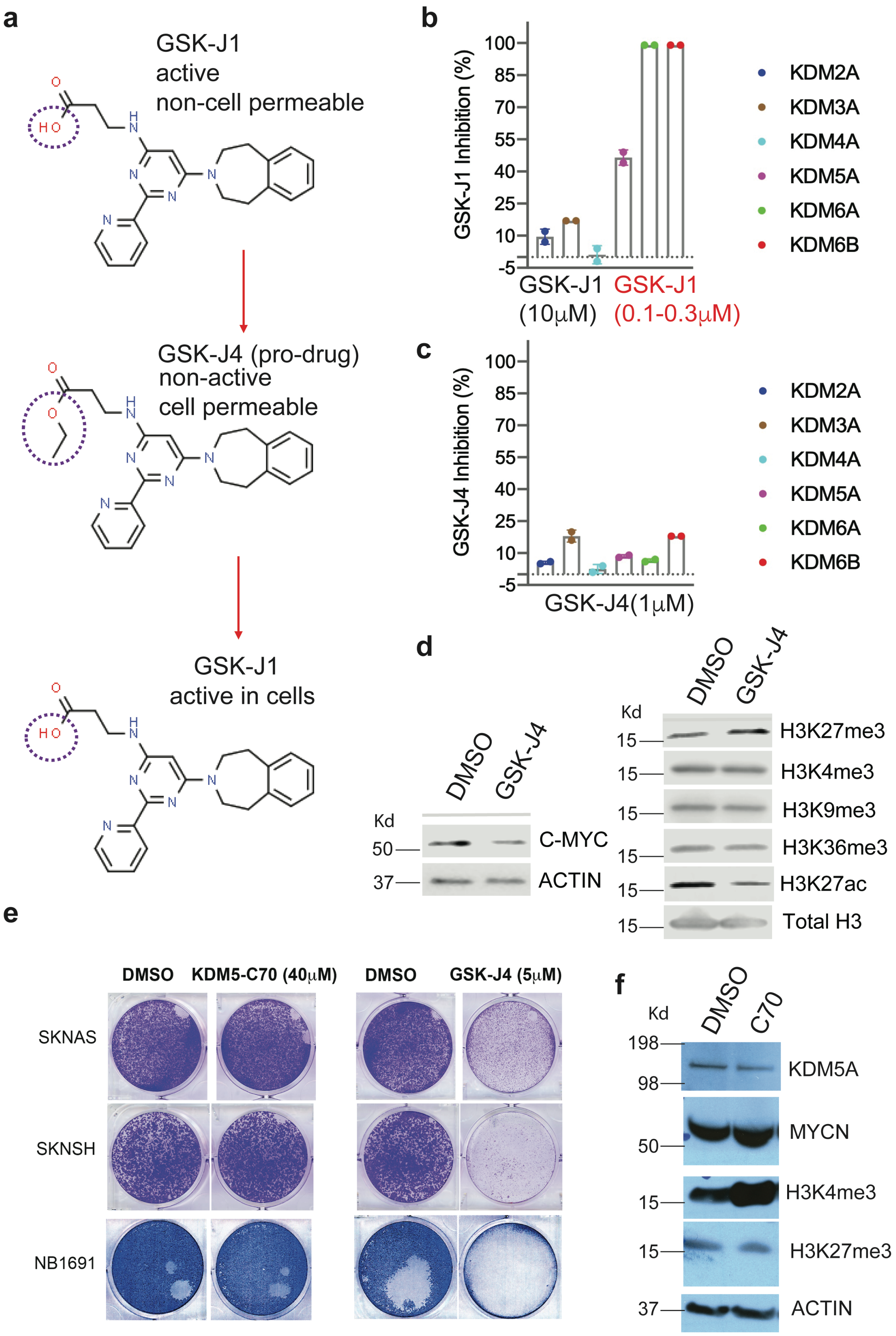
a
GSK-J1
active
non-cell permeable
GSK-J4 (pro-drug)
non-active
cell permeable
GSK-J1
active in cells
b
GSK-J1 Inhibition (%)
100
85
70
55
40
25
10
-5
GSK-J1 (10μM)
GSK-J1 (0.1-0.3μM)
KDM2A
KDM3A
KDM4A
KDM5A
KDM6A
KDM6B
c
GSK-J4 Inhibition (%)
GSK-J4(1μM)
d
Kd
DMSO
GSK-J4
50
37
C-MYC
ACTIN
15
H3K27me3
H3K4me3
H3K9me3
H3K36me3
H3K27ac
Total H3
e
DMSO
KDM5-C70 (40μM)
GSK-J4 (5μM)
SKNAS
SKNSH
NB1691
f
C70
198
98
KDM5A
MYCN
H3K4me3
H3K27me3
ACTIN

Supplementary Fig. 5 Profiling of GSK-J1 and GSK-J4 against KDMs. a GSK-J1, as a selective KDM6 inhibitor, is unable to penetrate cells. To make a cell-permeable inhibitor, GSK-J1 was modified by adding an ester (named as GSK-J4) specifically to enable efficient intracellular delivery of the compound. Once entering cells, GSK-J4 converts to GSK-J1 for inhibiting KDM6. **b, c** AlphasLISA applied to tests the inhibitory activity of GSK-J1 and GSK-J4 against purified KDMs in vitro, to validate the GSK-J1 selectivity against KDMs. 0.1 μM of GSK-J1 for KDM6A and KDM5A, and 0.3 μM of GSK-J1 for KDM6B. n=2 for each KDM. Data are represented as mean ± SD. **d** SK-N-AS cells were treated with GSK-J4 at 2.5μm for 48 hours. Cell lysates were subject to immunoblotting with indicated antibodies. The blots are representative of three independent experiments. **e** SK-N-AS, SK-N-SH and NB-1691 cells were treated with 40μM of KDM5-C70 or 5μM of GSK-J4 for 10 days, followed by crystal violet staining. **f** NB-1691 cells treated with 40μM of KDM5-C70 for 72 hours were subject to immunoblotting with indicated antibodies. The blots are representative of three independent experiments.

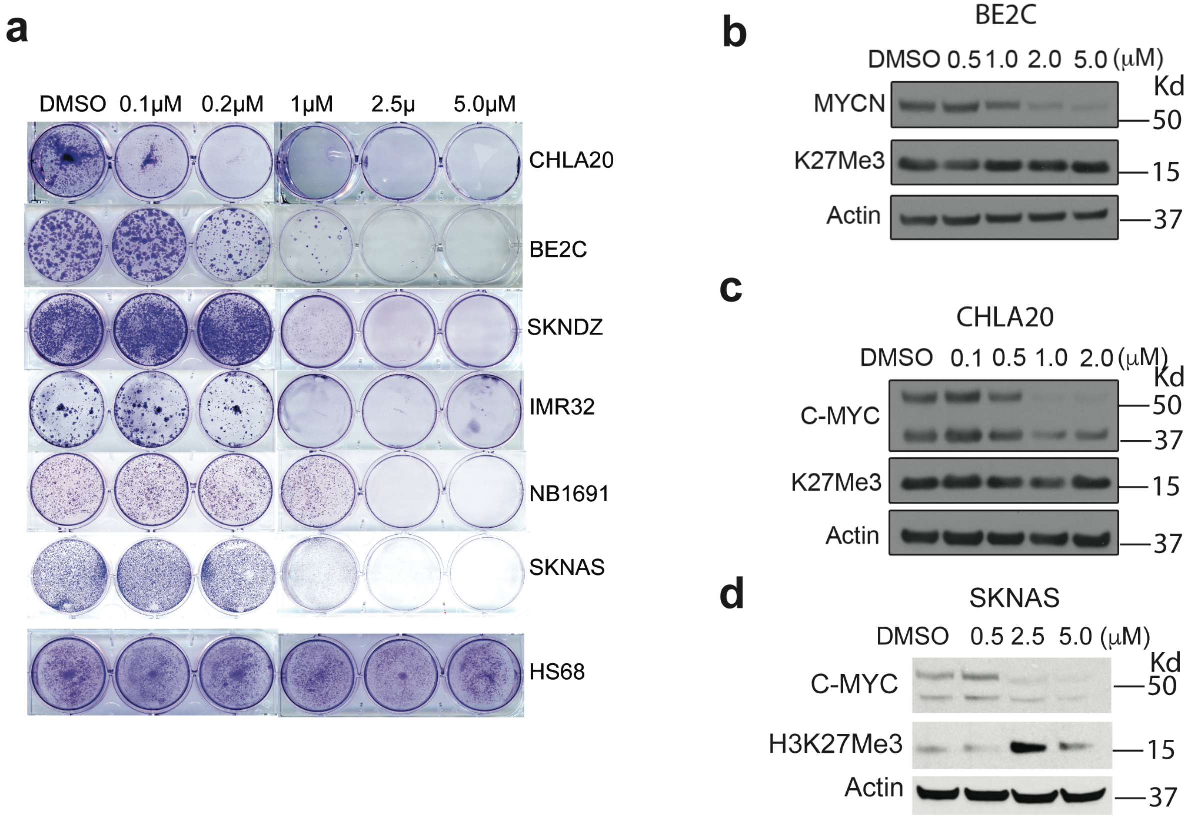

Supplementary Fig. 6 GSK-J4 targets MYC expression. **a** Crystal violet staining of colonies after neuroblastoma cell lines and human normal fibroblast HS68 cells were treated with different concentrations of GSK-J4 for 7 days. **b-d** Western blot analysis with indicated antibodies to assess MYCN or C-MYC and H3K27me3 expression after 48-hour treatment with GSK-J4 of BE2C (**b**), CHLA20 (**c**) and SKNAS cells (**d**). The blots are representative of three independent experiments.

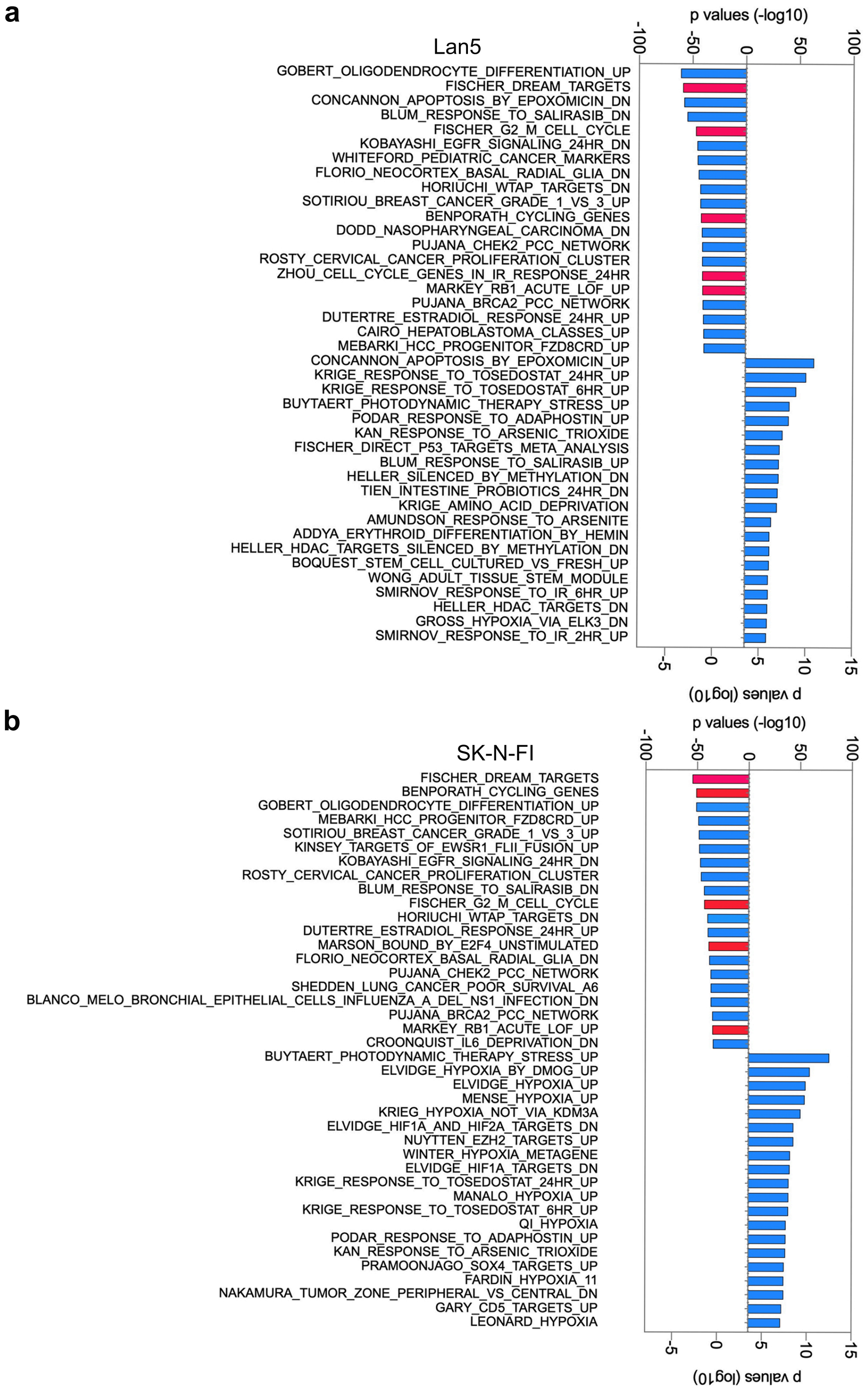

Supplementary Fig. 7 GSK-J4 inhibits E2F pathways. GSEA scores for the significant differentially expressed genes (downregulated and upregulated) induced by GSK-J4 in LAN5 and SK-N-FI cells[41] that were analyzed using GSEA online program (https://www.gsea-msigdb.org/gsea/msigdb/annotate.jsp) for pathway enrichment in database CGP: chemical and genetic perturbations. Red color indicates the gene sets of Rb1-E2F and cell cycle. P value calculated by one-sided Fisher's exact test.

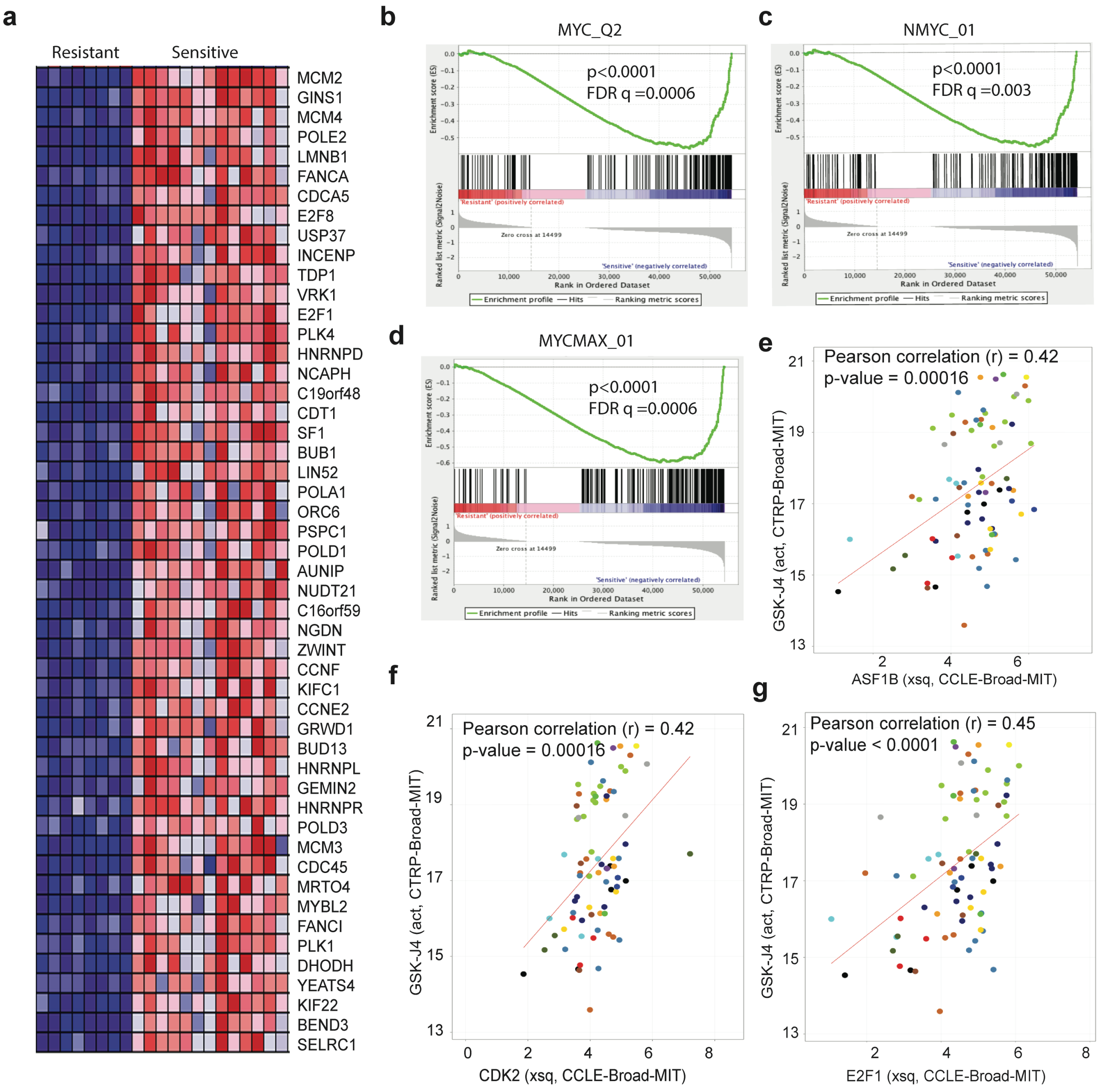

Supplementary Fig. 8 E2F signature is associated with GSK-J4 sensitivity. a The GSEA heatmap shows the top 50 genes highly expressed in GSK-J4 sensitive (group A) vs resistant (group B), after analysis of data which was extracted from The Cancer Therapeutics Response Portal (CTRP). **b-d** GSEA show that genes highly expressed in GSK-J4 sensitive (group A) cells are enriched with MYC gene sets. P value calculated by one-sided Fisher's exact test. The FDR is calculated by comparing the distribution of normalized enrichment scores from many different genesets. **e-g** The Pearson correlation (two-tailed) of GSK-J4 with expression of E2F target genes, ASF1B(**e**), CDK2 (**f**), E2F1 (**g**), analyzed by using the online program https://discover.nci.nih.gov/cellminercdb/.

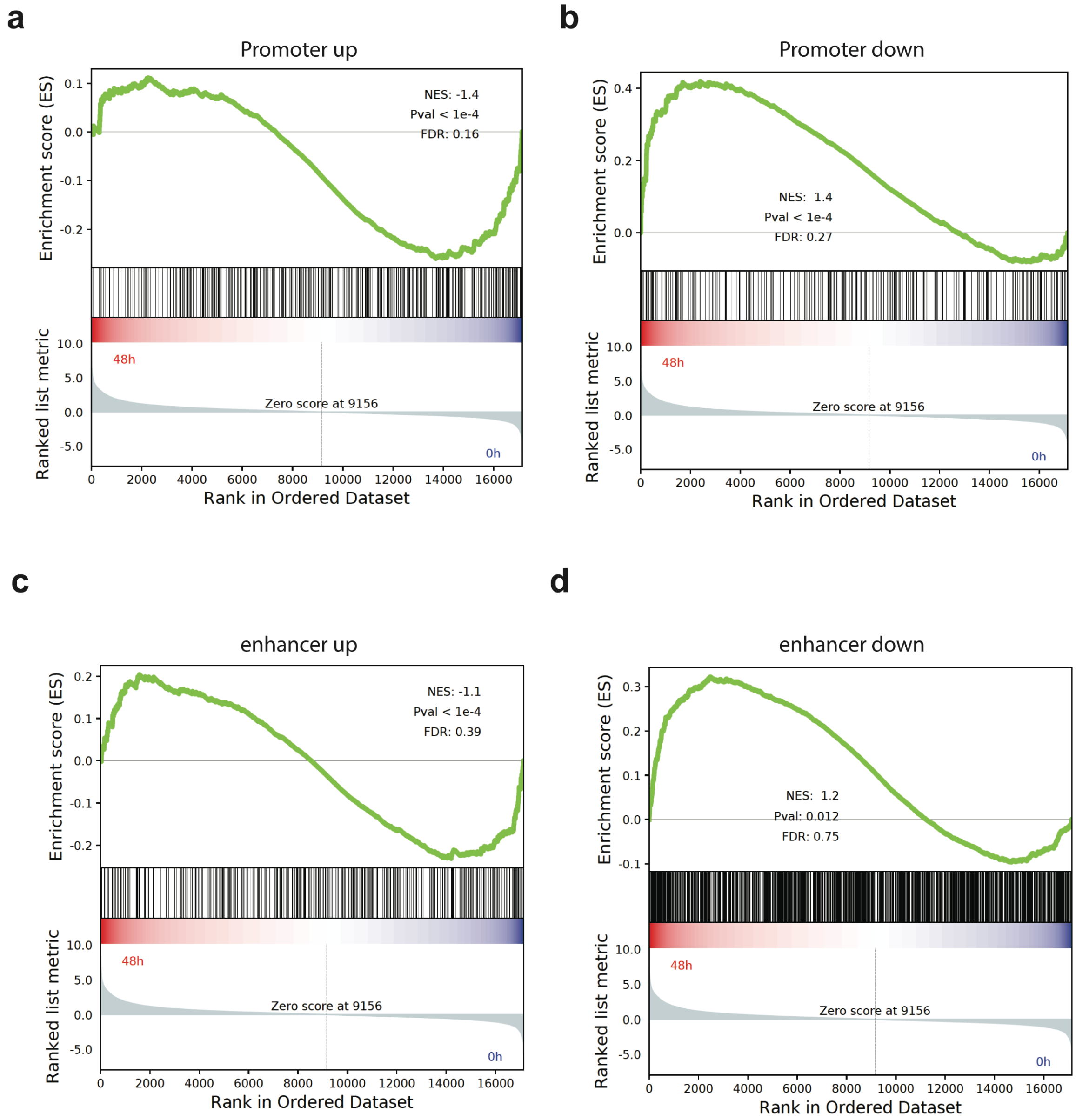

Supplementary Fig. 9. GSEA analysis of differentially altered chromatin accessibility regions against the gene expression data based on RNA-seq at 48h treatment by GSK-J4. P value calculated by one-sided Fisher's exact test. The FDR is calculated by comparing the distribution of normalized enrichment scores from many different genesets.

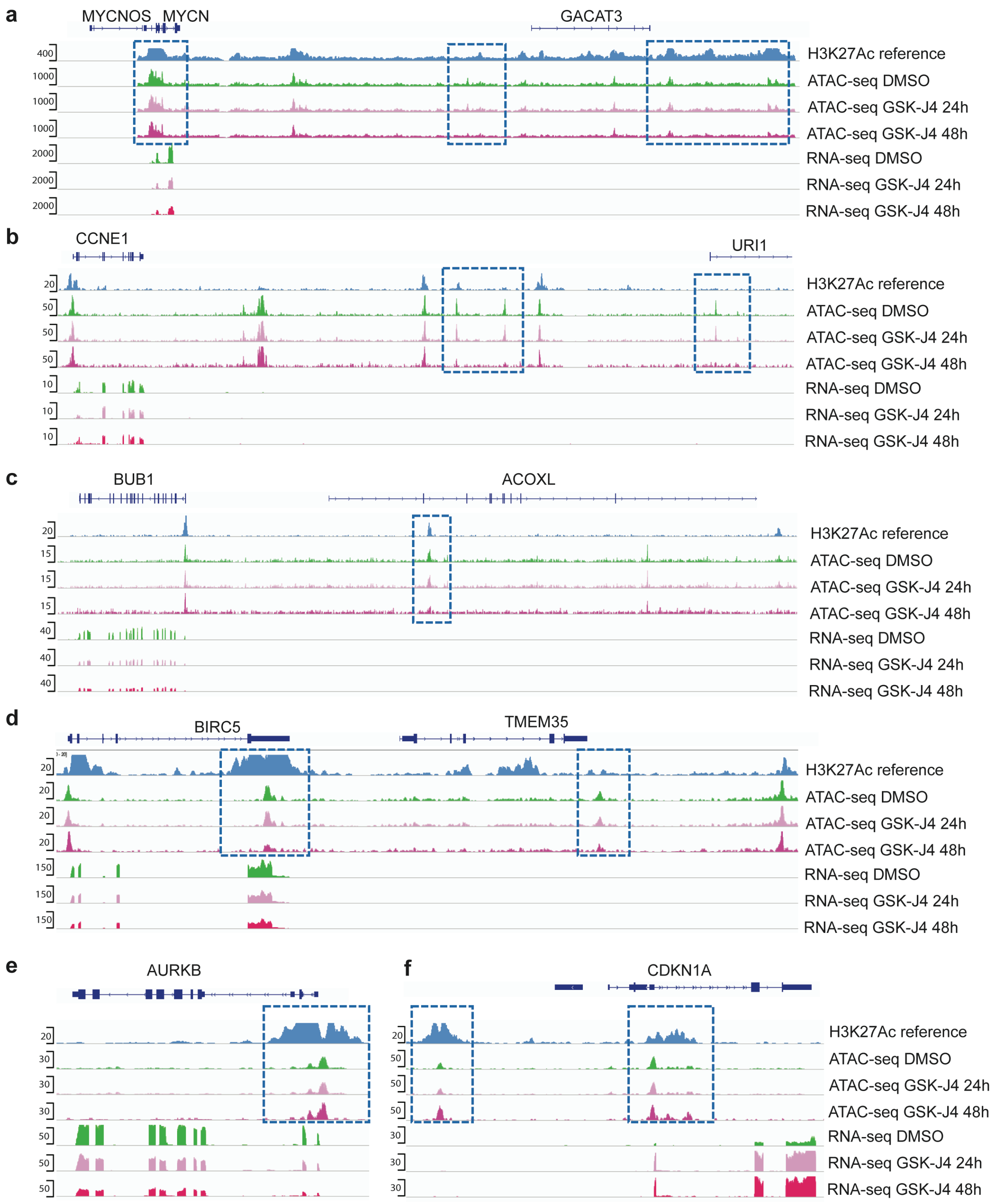

Supplementary Fig. 10. The ATAC-seq analysis of *MYCN* and cell cycle genes (*CCNE1*, *BUB1*, *BIRC5*, *AURKB*, *CDKN1A*) shows the alterations of DNA accessibility at their promoter and distal enhancer regions by GSK-J4 treatment for 24h and 48h. RNA-seq data analysis shows the RNA reads effected by GSK-J4 treatment for 24h and 48h.

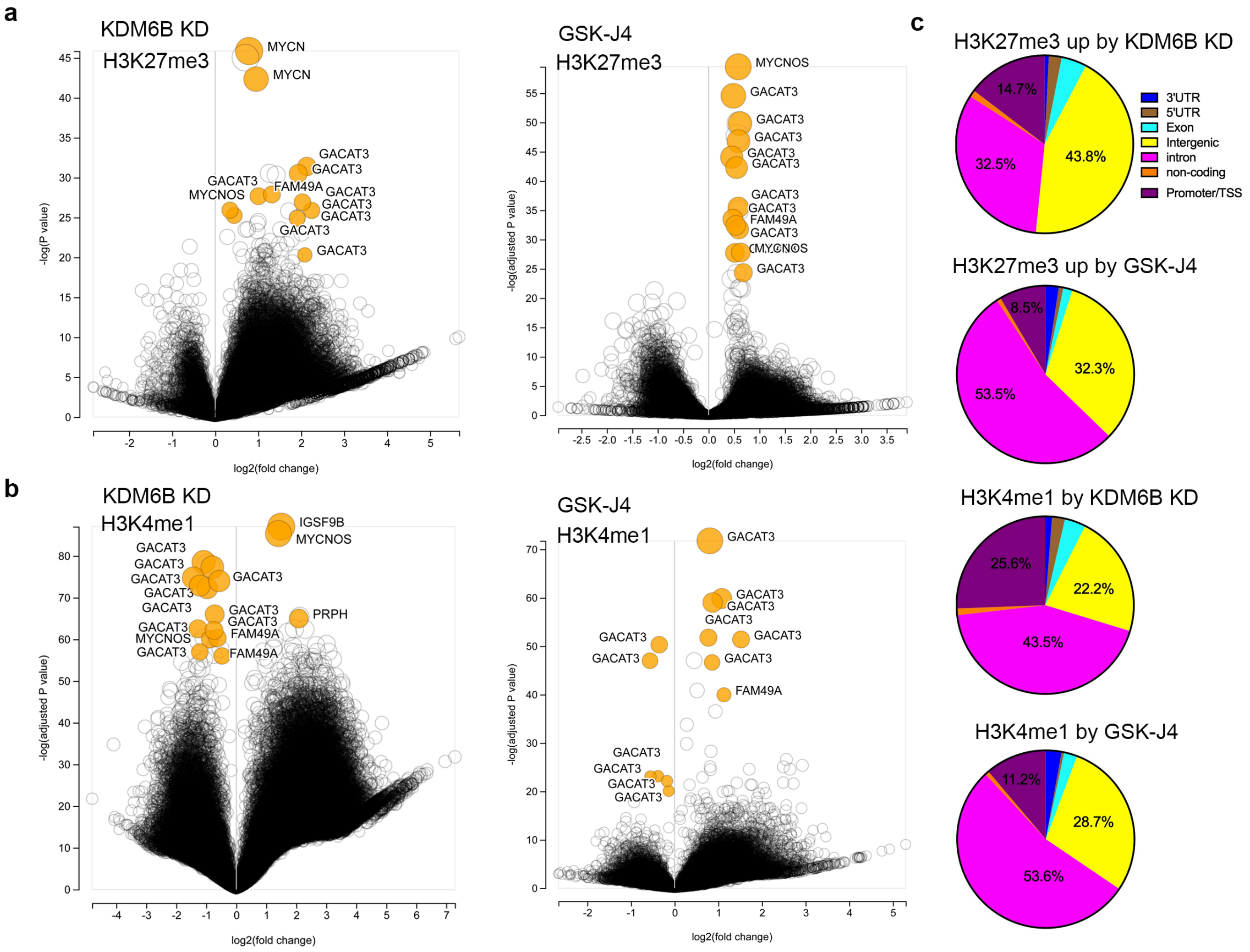

Supplementary Fig. 11. Impact of epigenetic marks by KDM6B inhibition. a, b Volcano plot showing differential peaks of H3K27me3 (**a**) and H3K4me1 (**b**) induced by KDM6B knockdown and GSK-J4 treatment of BE2C cells. The yellow color highlights the super-enhancer region of *MYCN*, GACAT3 locus. The y-axis indicates p value. The x-axis indicates fold change. The p-value is obtained from the limma moderated t-statistic, two-sided. **c** Pie plot showing the percentage of H3K27me3 upregulated by KDM6B inhibition and global H3K4me1 at distinct genomic regions.

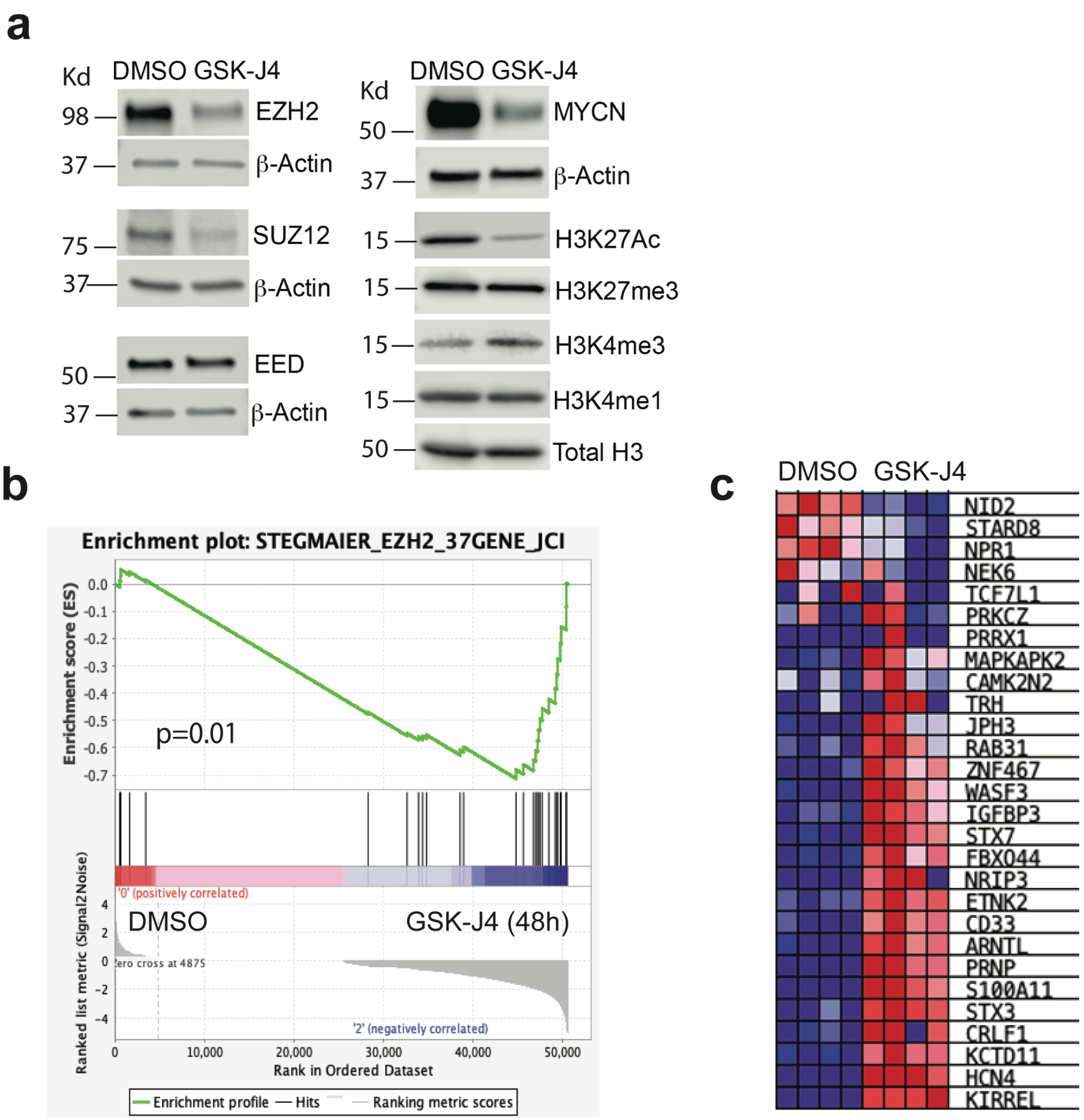

Supplementary Fig. 12 GSK-J4 downregulates the expression of PRC2 complex. a Western blot analysis with indicated antibodies to assess PRC2 complex, MYCN and histone modification mark expression after 48-hour treatment with 2.5μM of GSK-J4 of BE2C. The blots are representative of three independent experiments. **b** GSEA analysis of EZH2 signature against the gene expression data based on RNA-seq at 48h treatment by GSK-J4. P value calculated by one-sided Fisher's exact test. **c** Heatmap of the 37-gene signature from **c**.

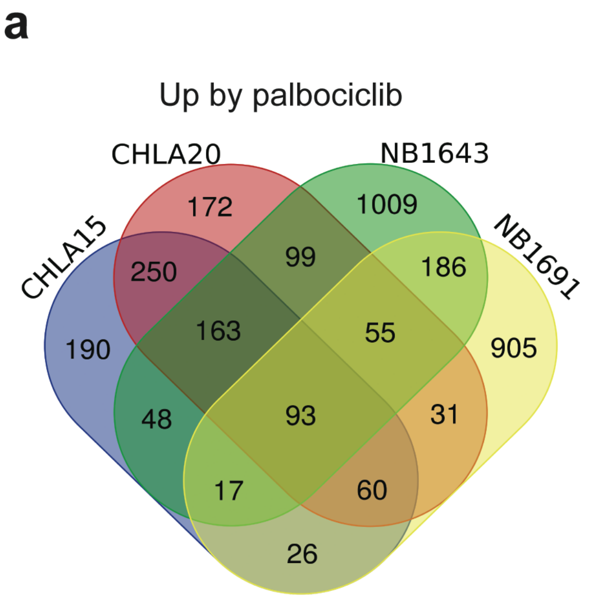

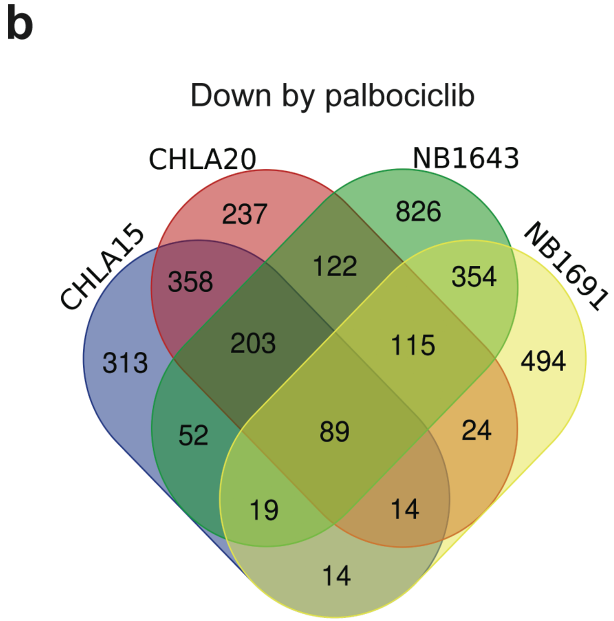

Supplementary Fig. 13 Common genes done regulated and upregulated by palbociclib. Four neuroblastoma cells lines were treated with palbociclib for RNA-seq. The differentially expressed genes in each cell line were analyzed by using Venn program to find the commonly upregulated (**a**) or downregulated genes (**b**)

a

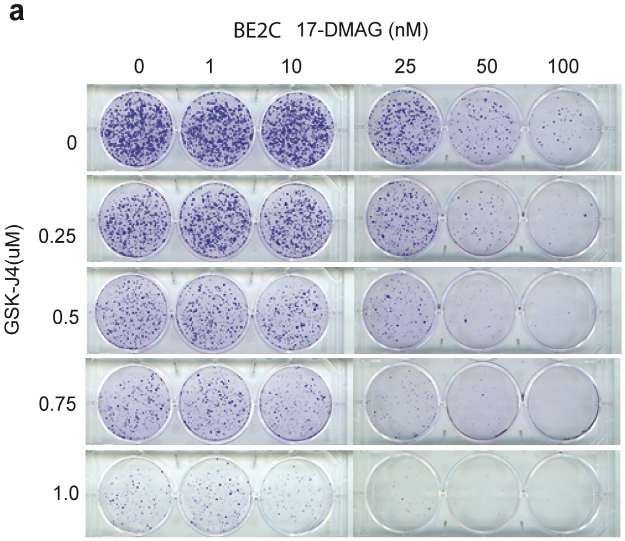

c

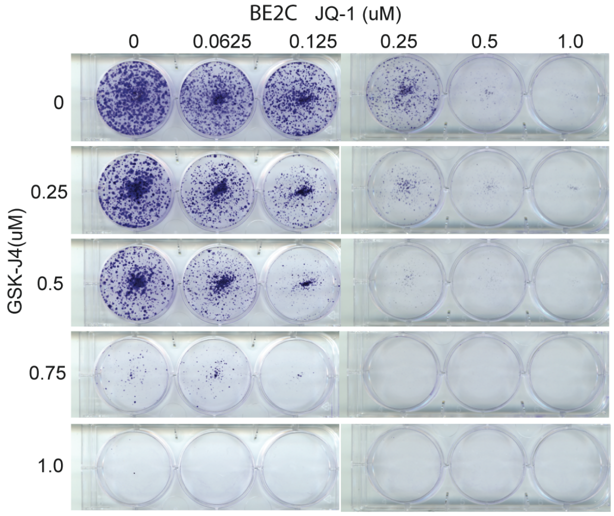

b

BE2C 17-DMAG (nM)

GSK-J4(uM) — Percent over bliss score	0	1	10	25	50	100
0						
0.25		-0.1	-1.5	1.5	3.1	7.3
0.5		7.9	0.5	5.4	12.9	12.0
1		1.7	9.7	9.7	6.8	4.8

d

BE2C JQ-1(uM)

GSK-J4(uM) — Percent over bliss score	0	0.0625	0.125	0.25	0.5	1
0						
0.25		3.5	3.5	4.7	5.7	6.1
0.5		5.3	6.5	9.0	8.2	10.2
1		-6.6	-2.6	-2.0	1.0	7.7

e

SK-N-AS Palbocicilb (uM)

GSK-J4(uM)	0	0.25	0.5	1	2	4
0						
0.5		2.8	0.7	0.5	-7.4	-1.3
1		1.9	0.1	-2.8	-2.3	2.7
2.5		-3.6	-2.3	-3.3	2.8	3.9

f

SK-N-AS 17-DMAG (nM)

GSK-J4(uM)	0	1	10	25	50	100
0						
0.5		5.2	-3.2	-6.4	2.3	2.0
1		-3.5	-6.2	-7.3	2.9	3.1
2.5		-2.8	-7.9	-4.7	1.3	1.9

g

SK-N-AS JQ-1(uM)

GSK-J4(uM)	0	0.0625	0.125	0.25	0.5	1
0						
0.5		-2.8	-1.4	0.8	-1.2	-3.8
1		-1.1	-3.4	-1.4	-7.0	2.1
2.5		0.6	2.1	-1.5	3.6	4.3

Supplementary Fig. 14 KDM6 inhibition is synergistic with non-CDK4/6 inhibitors. a BE2C cells were seeded with low numbers in 6-well plate and treated with different concentrations of GSK-J4 or/and 17-DMAG for 7 days. The cell colonies were stained with crystal violet. **b** Bliss index for combination of GSK-J4 and 17-DMAG in BE2C cells. Positive scores indicate synergy, negative scores indicate antagonism. **c** BE2C cells were seeded with low numbers in 6-well plate and treated with different concentrations of GSK-J4 or/and JQ-1 for 7 days. The cell colonies were stained with crystal violet. **d** Bliss index for combination of GSK-J4 and JQ-1 in BE2C cells. Positive scores indicate synergy, negative scores indicate antagonism. **e** Bliss index for combination of GSK-J4 and palbociclib in SK-N-AS cells. Positive scores indicate synergy, negative scores indicate antagonism. **f** Bliss index for combination of

GSK-J4 and 17-DMAG in SK-N-AS cells. Positive scores indicate synergy, negative scores indicate antagonism. **g** Bliss index for combination of GSK-J4 and JQ-1 in SK-N-AS cells. Positive scores indicate synergy, negative scores indicate antagonism.

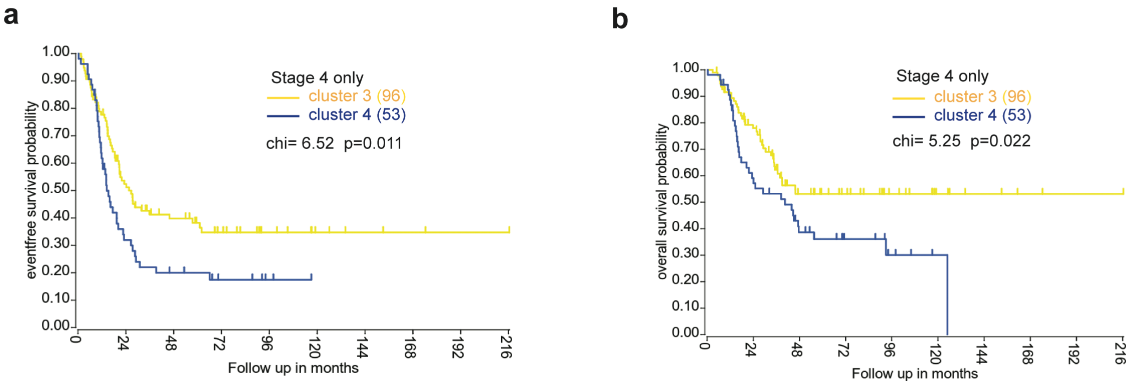

Supplementary Fig. 15 Kaplan-Meier curve using Log-Rank method shows the event-free survival and overall survival of clusters 3 and 4 with stage 4 disease.

Supplementary Table 1. Cell lines related to Fig. 3b.

Resistant cells	KYSE30_OESOPHAGUS
	TUHR14TKB_KIDNEY
	BICR56_UPPER_AERODIGESTIVE_TRACT
	HS940T_FIBROBLAST
	HS688AT_FIBROBLAST
	KMRC3_KIDNEY
	KG1C_CENTRAL_NERVOUS_SYSTEM
	HS822T_FIBROBLAST
Sensitive cells	KASUMI2_HAEMATOPOIETIC_AND_LYMPHOID_TISSUE
	HGC27_STOMACH
	KYM1_SOFT_TISSUE
	COV434_OVARY
	DMS53_LUNG
	A204_SOFT_TISSUE
	KMBC2_URINARY_TRACT
	T84_LARGE_INTESTINE
	MDAMB453_BREAST
	DMS114_LUNG
	KE39_STOMACH
	SKNBE2_AUTONOMIC_GANGLIA
	SNU216_STOMACH

Supplementary Table 2. Transcription factor motif (TFT) for group A cells in Fig. 3b.

Gene set enrichment scores analyzed by GSEA program. ES= enrichment score, NES = normalized enrichment score, NOM P-val = nominal p value. FDR =false discovery rate. P value calculated by one-sided Fisher's exact test. The FDR is calculated by comparing the distribution of normalized enrichment scores from many different genesets.

	Gene sets	ES	NES	NOM p-val	FDR q-val
1	E2F_Q3	-0.73	-2.27	0	0
2	E2F_Q4_01	-0.74	-2.24	0	0
3	E2F_Q6_01	-0.71	-2.24	0	0
4	E2F1_Q6	-0.77	-2.23	0	0
5	E2F1_Q3	-0.76	-2.23	0	0
6	E2F1_Q4_01	-0.72	-2.23	0	0
7	E2F1_Q6_01	-0.76	-2.22	0	0
8	E2F_Q3_01	-0.72	-2.22	0	0
9	SGCGSSAAA_E2F1DP2_01	-0.78	-2.21	0	0
10	E2F1DP1_01	-0.75	-2.21	0	0
11	E2F1DP2_01	-0.75	-2.21	0	0
12	E2F4DP2_01	-0.75	-2.21	0	0
13	E2F4DP1_01	-0.75	-2.21	0	0
14	E2F_03	-0.72	-2.21	0	0
15	E2F_Q6	-0.76	-2.21	0	0
16	E2F_02	-0.75	-2.2	0	0
17	E2F_Q4	-0.75	-2.19	0	0
18	E2F1DP1RB_01	-0.73	-2.19	0	0
19	KTGGYRSGAA_UNKNOWN	-0.71	-2.17	0	0
20	E2F_01	-0.76	-2.15	0	0
21	E2F1_Q4	-0.66	-2.11	0	0

Supplementary Table 3. Transcription factor binding motif enrichment for neuroblastoma cells sensitive to GSK-J4. Gene set enrichment scores analyzed by GSEA program. ES= enrichment score, NES = normalized enrichment score, NOM P-val = nominal p value. FDR =false discovery rate. P value calculated by one-sided Fisher's exact test. The FDR is calculated by comparing the distribution of normalized enrichment scores from many different genesets.

	Gene Sets	NES	NOM p-val	FDR q-val
1	E2F_Q6	1.89019	0.00000	0.00000
2	E2F_Q4	1.86916	0.00000	0.00112
3	E2F4DP2_01	1.84061	0.00000	0.00075
4	E2F_02	1.83652	0.00000	0.00112
5	E2F1DP1_01	1.83527	0.00000	0.00090
6	E2F1_Q6	1.83142	0.00000	0.00075
7	E2F1DP2_01	1.83124	0.00000	0.00064
8	E2F1_Q3	1.82484	0.00000	0.00056
9	E2F1DP1RB_01	1.80926	0.00000	0.00050
10	E2F4DP1_01	1.80680	0.00000	0.00045
11	E2F_Q3_01	1.80364	0.00000	0.00041
12	E2F_03	1.79745	0.00000	0.00064
13	SGCGSSAAA_E2F1DP2_01	1.79145	0.00000	0.00076
14	E2F1_Q4_01	1.77454	0.00000	0.00125
15	E2F_Q4_01	1.77275	0.00000	0.00117
16	E2F_Q6_01	1.71608	0.00000	0.00258
17	E2F1_Q6_01	1.67011	0.00000	0.00468
18	E2F_Q3	1.66943	0.00000	0.00448
19	KRCTCNNNNMANAGC_UNKNOWN	1.63733	0.00184	0.00603
20	NRF1_Q6	1.55445	0.00000	0.01859
21	NRSF_01	1.54447	0.00000	0.01968
22	TMTCGCGANR_UNKNOWN	1.52952	0.00000	0.02146
23	CAGNWMCNNNGAC_UNKNOWN	1.52403	0.00000	0.02186
24	ACTAYRNNNCCCR_UNKNOWN	1.51553	0.00000	0.02396
25	MCAATNNNNNGCG_UNKNOWN	1.51439	0.00372	0.02336

Supplementary Table 4. Genes downregulated by KDM6B KD related to Fig. 6a.

Description	log 2 (FC)	Description	log 2 (FC)	Description	log 2 (FC)	Description	log 2 (FC)	Description	log 2 (FC)	Description	log 2 (FC)
DBH	2.321632	TK1	1.137371	ASAP1	0.964286	HMBS	0.864758	B3GALT2	0.783497	HPRT1	0.732571
FAM111B	2.211132	NCS1	1.137318	BNIP3	0.9637785	COL6A6	0.8639975	DLL3	0.7830965	CAMKK1	0.731661
ADGRG2	2.060494	TAGLN2	1.1365735	RFK	0.963643	ST3GAL4	0.86233	KCNA3	0.782769	SLC2A6	0.7314655
RTL1	1.950828	PPP1R16B	1.135968	LRRTM1	0.9628975	PSMD11	0.8599885	ZFP36	0.782336	CIART	0.7305685
CD9	1.925528	NETO2	1.135297	LGR5	0.96257	SLC2A6	0.859512	GTSE1	0.782211	CBWD6	0.7292315
OAS1	1.8900065	MID1	1.1349535	RHOB	0.9624545	EBAG9	0.859349	GP1BB	0.781957	RFC2	0.7288095
GNL2	1.836948	S1PR1	1.133851	SLIT3	0.9622515	SYT9	0.859347	CCP110	0.781763	ITGA9	0.7288065
KDM6B	1.8246645	ESCO2	1.1333755	MYB	0.9621145	HJURP	0.8593015	SMCO4	0.781604	NUSAP1	0.727813
ASS1	1.7042745	CDK2	1.133259	PFN2	0.960925	SLC6A17	0.858509	KCNH4	0.7806445	POLA1	0.7273485
ACE	1.7006315	GSTT2	1.1321125	CLVS1	0.9587585	MIF4GD	0.8575665	TCF3	0.780139	TCF7L2	0.7271315
CACNG4	1.667475	EXOSC1	1.1308815	AS3MT	0.9583305	ATAD2	0.856628	MIR6809	0.779837	DNAAF5	0.727124
CCNE2	1.6435515	ARHGAP29	1.125485	TLCD1	0.9577255	OSBPL3	0.85603	FAM105A	0.7789145	BORA	0.7269125
OLFM1	1.6381345	CEP295NL	1.124688	B3GALNT1	0.9570825	HMBS	0.855616	C11orf24	0.7782945	SYN2	0.7268225
C1QL1	1.6323405	PAPOLA	1.122019	ISYNA1	0.9567885	POLD3	0.8555495	PLEKHA6	0.778292	IDH2	0.726172
SH3BGRL3	1.5694855	NTAN1	1.11968	HIST1H4I	0.9552375	STK17B	0.853299	NDC80	0.7782305	EIF2B2	0.7258495
ADGRG1	1.550942	MTMR6	1.111176	MYBL2	0.954745	FABP5	0.8517465	ARHGAP42	0.7780475	NR2C2AP	0.725077
CLSPN	1.5505425	BEGAIN	1.1093	DHFR	0.954631	PWP2	0.849862	ZWINT	0.777092	CD3EAP	0.722806
ZMAT4	1.548271	ITPRIPL1	1.106137	CARHSP1	0.954499	DLGAP5	0.8496545	AIFM2	0.776263	FANCI	0.722727
CHRM1	1.546421	TUBB3	1.1050985	RAD51	0.9543475	RNASEH2B	0.8493805	CENPA	0.774866	SLCO3A1	0.722494
ASCL1	1.504814	CENPW	1.1012415	RD3	0.953663	HIST1H2BJ	0.8477665	FOXRED2	0.7743005	DDAH1	0.720853
PLPP4	1.5006365	PRR11	1.0977845	ALKBH5	0.953464	LRFN2	0.847708	ZCCHC10	0.7734935	FUZ	0.7205335
LSM6	1.4959865	CDC42BPA	1.095631	CENPI	0.9531245	TRMU	0.847394	EFCAB10	0.77289	CD151	0.7199095
DRD2	1.4847435	RAB30	1.092287	SLC2A6	0.952743	HIST1H2BE	0.845258	MIR17HG	0.772691	HAUS1	0.7195635
S100A11	1.4830285	TPM1	1.08898	EZR	0.952128	HIST1H3F	0.8440505	CCNA2	0.7723315	LPCAT2	0.7194905
RELL1	1.458951	DSEL	1.088601	MRM1	0.951594	SS18	0.844034	NUDCD1	0.7723295	FARP1	0.7192205
PCDH19	1.4581215	C8orf88	1.0848885	WDHD1	0.9501385	CDKN2C	0.843517	DDX39A	0.7721355	LPAR3	0.7187545
FAM167A	1.458081	RPH3A	1.084791	KCNH5	0.948129	OGFRL1	0.8424345	FBXO33	0.771985	CCNB2	0.7187045
TSPAN18	1.452157	GLIPR2	1.08398	HIST1H2AB	0.94807	UBE2T	0.8421725	HIST1H4D	0.771535	DHRS11	0.7184735
TRPA1	1.442992	PLCB3	1.0823785	CASP7	0.9458635	THAP10	0.841529	MCM4	0.771309	ACTN4	0.7183585
CYB561	1.4197795	HIST1H1D	1.080605	CHAC2	0.9443185	KCNH2	0.841467	FOXM1	0.7705755	HDGFRP3	0.718223
EGR1	1.4148115	SFXN3	1.080087	SLC29A1	0.9430425	ENDOG	0.8396315	CSRP1	0.770482	FAM103A1	0.7156825
DDC	1.4067025	PPP2R2B	1.0777015	SLC20A1	0.9411075	LIFR	0.8396285	KLF6	0.769967	ADRA2C	0.7155855
ADAMTS1	1.401651	LOXL1	1.0775	NCKAP1	0.940983	MZT1	0.8384925	MED15	0.7687735	THSD7A	0.7143555
ATF3	1.39639	DSE	1.076164	C1orf21	0.939349	GATA2	0.8363735	SYNPO	0.767908	EMC8	0.7114695
FXYD6	1.391147	VGF	1.074699	LTA4H	0.9381635	ODAM	0.835778	CDCA7	0.767407	CENPK	0.7112845
HGF	1.3783205	CORO1A	1.0743285	E2F7	0.9378295	PRIM1	0.835587	CNTFR	0.767388	ITGA6	0.710897
LPGAT1	1.377573	MMD	1.0737455	HIST1H3I	0.937742	FAM72A	0.835125	MSH6	0.7673295	MYCN	0.71007
PARM1	1.358232	NCAPG	1.071829	EXOSC7	0.9374075	MASTL	0.835066	CDC20	0.767083	H2AFX	0.710038
RIMS3	1.355447	NR1D2	1.0714375	MARCH4	0.934672	LRR1	0.8343785	GEMIN5	0.7666695	LIG1	0.7099835
E2F8	1.354234	FEN1	1.071426	CENPE	0.934277	ALCAM	0.8316485	TRIM29	0.7662365	ST8SIA2	0.7099395
CHST11	1.3470885	ADCYAP1	1.0713965	VTI1B	0.9326895	HES1	0.8299475	RNF138	0.7661765	ENPP2	0.7097335
SLC18A1	1.3455875	KIF20A	1.0709125	CNN3	0.931465	MSI2	0.8297345	SLC2A6	0.7645365	SH3RF1	0.7094135
HTR3A	1.344718	FBXO5	1.0696375	CDKN3	0.931385	VARS	0.8282165	KDELR3	0.764033	SYT4	0.708528
GRIK3	1.3440255	HIST1H2BI	1.069534	EAF1	0.930857	PTGER2	0.828158	HACD1	0.763019	DNA2	0.7078805
GABRA4	1.340389	HIST2H3A	1.068472	CAMKV	0.9292875	MAGI1	0.827905	CALM3	0.7626735	SYNPO2	0.7058915
CORO2A	1.3199035	WDR76	1.0669695	FUNDC1	0.928694	GPATCH4	0.827681	ETS2	0.7612885	ANP32E	0.705295
SPRY4	1.313135	NRG1	1.066745	HIST1H3G	0.9280345	KCNT1	0.8274855	CRABP2	0.7610885	CHGA	0.7040255
ENTPD3	1.312376	CLN6	1.057853	HEG1	0.9273205	DUSP7	0.8273865	COL10A1	0.7608495	NR2F2	0.70384
CRIPT	1.3078695	GPR3	1.0517225	DSN1	0.926089	PCBP4	0.827072	5-Mar	0.760535	CRABP1	0.703754
PPEF1	1.306663	HIST1H3B	1.0510495	PHLDA1	0.925801	SPIN4	0.8262735	RPA3	0.760497	SHC1	0.702837
HIGD1C	1.3061335	KCNQ2	1.0509405	HS6ST1	0.924628	LAMP5	0.82597	PLAT	0.760285	HIST1H2AJ	0.702205
DGKK	1.3045375	HIST1H2AE	1.0494565	CREB5	0.924266	FAM26E	0.8254605	KCNH6	0.7583625	TMED5	0.701647
TFDP1	1.302833	CHAF1B	1.0485785	CDC25A	0.918888	HSPB1	0.8247935	TNFRSF19	0.7582825	NPY2R	0.7012725
SULF2	1.3026105	GSTT2	1.045235	PCSK1	0.917031	FLT1	0.824592	ABCB6	0.758199	ERI1	0.7004865
CDC7	1.2951595	DPY19L1	1.0440825	DDX21	0.9158435	EMP3	0.8238225	RAB33A	0.758004		
SQLE	1.295074	CAPN6	1.0422875	ARHGAP36	0.913221	MCM10	0.823391	ATP6V1E2	0.7579695		
HIST1H2BM	1.293814	GALNT6	1.0419675	CRIP1	0.912751	GNAZ	0.822219	MKI67	0.7579305		
FAM107B	1.290697	TH	1.040307	BORCS7-ASMT	0.90979	CXXC5	0.820824	DSCC1	0.757059		
LGALS1	1.2886055	DCLRE1A	1.034652	UCP2	0.908605	ALDH1B1	0.819841	G6PC3	0.756923		
SERTAD4	1.287473	HIST2H3A	1.034609	DNPEP	0.907702	FAM72B	0.8189115	DPP7	0.7560065		
GAL	1.28564	HPCAL1	1.031487	SLC25A43	0.907002	PWP2	0.818265	CDK2AP1	0.753725		
P2RY6	1.2848965	PTP4A2	1.029474	HIST2H2AB	0.9066315	MAPK8IP1	0.817379	RABEPK	0.75363		
RPL22L1	1.2824695	PRSS3	1.0262955	MYO19	0.906319	DARS2	0.8169945	ATP1B1	0.7533895		
KCNJ8	1.2717995	AURKB	1.023682	LYN	0.903675	CEP55	0.8161995	KIF26A	0.7531245		
HS3ST2	1.2673875	UBE2C	1.022596	MELK	0.902073	HIST1H2BL	0.815815	LDB2	0.752315		
CDCA3	1.26694	FAM27E3	1.0208725	TPBGL	0.9018495	MTFR2	0.815646	SLC25A19	0.752212		
RRM2	1.2647935	MCHR1	1.0187285	CDKN1A	0.8985085	CLGN	0.814457	USP1	0.7519085		
CDC42EP3	1.2616845	IFT57	1.016883	TOB1	0.898062	LMNB1	0.813918	KLHL23	0.7502055		
LMNA	1.251905	KPNA3	1.016815	CDK1	0.897526	PRSS2	0.8138965	DTL	0.750125		
S100A10	1.243042	PSRC1	1.0144755	FAM83D	0.8953035	PLXNA2	0.813307	TIMP3	0.7497825		
CYSLTR2	1.2321625	SUZ12	1.0142565	HTR3B	0.8948555	CRH	0.813196	TUBB8	0.749779		
HELLS	1.2296565	CSRP2	1.013496	SCOC	0.893171	HIST1H3A	0.812526	CHML	0.7494815		
KIAA0125	1.227768	HNRNPH3	1.01341	GNB4	0.892913	EIF4EBP2	0.81247	FAM57A	0.7492065		
BMP2K	1.2217075	DIAPH3	1.010338	JUN	0.890321	HNRNPDL	0.81157	CD58	0.7484855		
TGFB1	1.2160885	GMNN	1.0079265	RMI1	0.8901375	GRAMD1A	0.810821	PAQR8	0.7484015		
TYMS	1.2151295	BAALC	1.007485	SNRNP27	0.889695	TTYH3	0.810707	CCNB1	0.7472795		
CDC45	1.2086675	MPHOSPH6	1.0042295	IER2	0.889119	SLC18A2	0.809766	RET	0.747065		
PSMC3IP	1.2026625	NPTN	1.0018175	FAM72C	0.8886445	ANGPTL2	0.8093625	DEK	0.7468275		
FRMD3	1.2021815	HIST3H2BB	1.001207	PPFIBP2	0.888034	DPF1	0.8089575	RND3	0.7439775		
DCK	1.2005485	KNTC1	1.000174	HIST1H2BF	0.887842	PIH1D1	0.8068015	MCM6	0.743682		
NSG1	1.181681	ENC1	1.000114	MCUR1	0.88769	RELN	0.8053735	ADAMTS7	0.7430355		
ELAVL3	1.179775	NRM	0.997943	RMI2	0.8859815	MCM2	0.805226	PNP	0.742527		
SLC25A32	1.176584	SH2B3	0.997209	LSM1	0.8837495	ENOPH1	0.805192	HIST1H1B	0.74043		
CORO1C	1.1704935	TLE3	0.995669	SRSF1	0.8828955	EXOSC5	0.8037515	NCOA3	0.7402835		
UHRF1	1.159782	PCNA	0.99512	CDON	0.8819025	TUBB4B	0.803145	PLOD2	0.7400575		
NXT2	1.1567385	MBNL3	0.9938415	SEH1L	0.8801325	TIPIN	0.801921	CXCR4	0.739905		
GFRA2	1.15497	NDUFAF6	0.990284	HIST1H1C	0.8796435	TCF12	0.8014205	GRAMD4	0.739758		
RGS16	1.1508545	EXO1	0.989692	DOK4	0.87874	POPDC2	0.8002575	CHEK1	0.738866		
LRRC34	1.1499455	DUSP4	0.9866795	PLEKHA6	0.8783175	RNF8	0.800197	CDCA5	0.737542		
MRAP2	1.148787	KIAA0101	0.9857315	PHF10	0.877968	ISLR	0.7997445	MCM8	0.7374675		
FAM222A	1.1487165	PRELID1	0.9852385	HIST1H4L	0.8771565	CTNNAL1	0.799674	CCDC74B	0.7367225		
KLHL23	1.147348	CDT1	0.983783	LAYN	0.876545	LRP8	0.7988175	3-Mar	0.736406		
SPAG5	1.14667	FTSJ3	0.983726	SHMT2	0.875315	SFXN4	0.795168	JUNB	0.7356215		
FAM35A	1.144537	FTSJ1	0.982525	PRDM1	0.872831	MAML2	0.793564	UAP1L1	0.7356215		
ICAM2	1.141429	IQCJ	0.9766395	MMS22L	0.871546	AMD1	0.79222	SEC24D	0.7351695		
TXNRD1	1.141068	DHFR	0.9721825	FAM72D	0.8692625	TUBG1	0.79183	ANAPC15	0.734412		
RCC1	1.140819	ALG13	0.9705435	AAK1	0.867935	ARHGEF28	0.7901005	PPP1R14B	0.733991		
TCF19	1.1399895	IGDCC3	0.9696875	SPSB4	0.8678725	TBL1XR1	0.7861465	ST6GAL1	0.7333735		
PPARGC1A	1.138357	LOX	0.964423	ETV4	0.8674795	MIS18A	0.7851315	PSAT1	0.7328895		
TK1	1.137371	POLE2	0.964339	STRA6	0.866528	XRCC2	0.7835765	HIST1H3D	0.7328775		

Supplementary Table 5. Genes downregulated by GSK-J4 related to Fig. 6b.

Description	log2 (FC)	Description	log2 (FC)	Description	log2 (FC)	Description	log2 (FC)
GABRA4	2.002878	CETN3	0.980721	IFIT5	0.8291315	HOMER1	0.7665685
GPR22	1.960247	EEF2KMT	0.9802005	VSTM4	0.82664	ANXA6	0.7662745
CDCA3	1.7204595	PPEF1	0.9784665	HSD17B7	0.8264445	CAPN6	0.7662495
CDK15	1.6739825	TUBB8	0.9718005	DLEU2	0.825846	DEPDC1B	0.765342
FAM72B	1.6477365	KIAA1024L	0.9716115	ZRANB2	0.824392	LCMT2	0.765181
FAM72A	1.5706995	HIST1H3A	0.967069	UNG	0.82302	KIF4A	0.764856
OAS1	1.5681005	ISOC1	0.96462	TIAM1	0.822294	HIST1H4D	0.7637735
HS3ST2	1.54878	NABP1	0.962395	DCTPP1	0.8213435	HIST1H2BB	0.763099
ARHGEF39	1.5020165	LRRC34	0.9586415	PRMT6	0.821043	CBR4	0.763014
FAM72D	1.4797305	KIF14	0.9562595	UCP2	0.820882	BORCS7-ASMT	0.7629915
BUB1	1.4796205	GNB4	0.956252	WDR46	0.820758	ALG1	0.7595085
CEP55	1.473713	SELENBP1	0.953799	KIFC1	0.820184	MKI67	0.758077
FAM72C	1.446828	MCM10	0.9522335	MARVELD1	0.8201745	ZNF287	0.7579165
ABCA12	1.3988565	GLA	0.9469565	CAB39L	0.8201555	HYLS1	0.757493
CENPA	1.3631	FBXO43	0.946167	CD47	0.819867	BUB1B	0.755509
PIGW	1.3570155	RMI1	0.9404065	MAGI1	0.8197065	CENPW	0.755419
CHAC2	1.355461	TNFAIP8	0.9389385	C3orf14	0.8196045	SCARA3	0.754553
DHCR24	1.3536805	S1PR1	0.9381585	CCDC167	0.8191785	RBPMS2	0.7528355
CDKN3	1.3530515	MPHOSPH6	0.9378775	METTL7A	0.81756	GTSE1	0.752401
POP5	1.3423625	CROT	0.9375545	HIST1H2BG	0.8138175	CTPS1	0.752385
ADAMTS1	1.3422115	NEFH	0.934242	C1orf131	0.8110815	FAM111B	0.751711
KIF20A	1.339638	ESCO2	0.929226	LRP8	0.8110585	PLK1	0.751675
SLC27A6	1.29907	LUM	0.9232585	CCNE1	0.8109765	CCNF	0.751412
LRRTM1	1.294278	COL6A6	0.9227425	CDCA2	0.810786	PNO1	0.7513895
ADAMTS4	1.290066	LHX9	0.9222	E2F8	0.8105455	FAM173B	0.750627
FABP5	1.2832225	HIST1H4L	0.922108	LRR1	0.809842	CCDC121	0.7488295
TTF2	1.2813805	CENPI	0.9201795	HIST2H3A	0.809542	SLC35E3	0.746751
HMMR	1.269343	TIPIN	0.917628	ABCD2	0.8094315	KIAA0125	0.7460965
HIST1H2BI	1.250498	BCKDHB	0.915872	EHD4	0.8092155	WDR35	0.7456365
SCGN	1.250291	AOX1	0.9152305	PALMD	0.8088365	FDPS	0.744777
PSRC1	1.232345	TUBD1	0.9009015	SOSTDC1	0.8083645	ANLN	0.7432525
SSPN	1.2238975	RPH3A	0.900191	LMNB1	0.8081145	CCNE2	0.7426865
FAM83D	1.2079625	POPDC2	0.8983815	MTERF3	0.8054075	ELAC2	0.740663
CCNB1	1.205968	PON2	0.8967725	POLE2	0.8049455	NUDT19	0.739674
PCDH18	1.202608	AURKA	0.896661	TTC38	0.8024935	CDC20	0.737603
SHOX2	1.18655	MTUS2	0.896244	SEMA3A	0.8014545	MCCC2	0.73455
HIST1H2AB	1.182082	RBM45	0.894017	KDM8	0.8010355	CBLN1	0.734272
SGOL2	1.1751515	GATS	0.8922285	C10orf128	0.8008925	LPCAT3	0.7335965
WDR77	1.173651	CDK1	0.890077	SLC25A53	0.7989435	CTSA	0.733365
TRIM59	1.1730225	TUBB4B	0.889865	ENTPD3	0.798145	GNG12	0.7326305
DLGAP5	1.1710225	PIK3R3	0.887125	DCAF6	0.798085	DPYSL5	0.731481
ADAM12	1.1449395	OIP5	0.8831055	AS3MT	0.797713	NEXN	0.7314255
TRMU	1.143721	CD3EAP	0.8818075	HIST2H4A	0.7970625	GRWD1	0.731416
HSPA2	1.140671	HIST1H1B	0.881675	EID1	0.795116	NUDCD2	0.7312255
DKK1	1.139863	RNF145	0.8802055	LIG4	0.7949885	BCCIP	0.7305175
ACE	1.1212455	ELK3	0.879889	RBM14	0.7941745	MTERF2	0.7303635
POLR3G	1.1188425	ECT2	0.874101	STOM	0.7938595	FJX1	0.730258
MRAP2	1.1010955	NOL6	0.870661	ITPRIPL1	0.793361	CCDC71	0.7296725
ZC3H4	1.094208	HSPA4L	0.869473	ITGA9	0.793001	KNOP1	0.7270215
HMGN5	1.0824625	CRYZ	0.868288	POLR3H	0.792142	NUSAP1	0.726642
POP1	1.077091	SRSF2	0.867641	C1QL1	0.791371	CLC	0.7237075
FRZB	1.0750525	GTF2H2	0.866721	ALDH4A1	0.7908575	HN1L	0.7234235
FABP5	1.0652625	NRP2	0.8664605	TMPO	0.7905385	TIMM8B	0.72046
RNF26	1.059643	ADI1	0.866282	ZDHHC16	0.7896755	CHCHD7	0.719318
TMEM19	1.0553845	NT5E	0.8653925	NDUFA10	0.7894015	ZNF480	0.7186005
TACC3	1.051755	TRIP13	0.8617965	PRDX1	0.7890845	MRPL50	0.7181995
WDR4	1.049956	EFHC2	0.8588445	UBE2S	0.7886335	RHOB	0.71716
CPT2	1.0412195	HELLS	0.858723	DCN	0.787255	GLRX2	0.7168405
HIST1H2BM	1.02962	RGS2	0.858473	GCH1	0.7869755	HIST1H2BJ	0.7135355
LCP1	1.0275845	AURKB	0.858079	INCENP	0.785728	CHTF8	0.7134925
C8orf48	1.024732	HIST2H3A	0.8557945	SEMA3E	0.7843485	UTP20	0.7129835
PTTG1	1.024696	POLR2L	0.85529	GMNN	0.782422	SLC2A6	0.712346
SLC46A3	1.0245685	RTL1	0.8526685	UBE2T	0.7808795	HRH3	0.710794
CENPE	1.0209985	KIF23	0.8524295	CD248	0.780732	GPSM2	0.710195
MBOAT4	1.0162175	SST	0.850459	RGL1	0.7800075	RBM14-RBM4	0.7094855
HTATIP2	1.012118	BEX1	0.8476705	SPDL1	0.779799	PUS7	0.709147
ACAT2	1.011124	RNFT1	0.8471105	DBI	0.7794305	CCNB2	0.7089505
IGFBP7	1.0103975	MAP2K6	0.8454915	P2RY6	0.7791215	GTF3C6	0.708847
PPP1R10	1.0065005	CLMP	0.841766	NLRP14	0.7779535	UBE2C	0.708718
SNRPG	1.0051075	RARG	0.8412255	FKBP7	0.777402	FAM217B	0.707986
SDHAF3	1.0047995	RAB29	0.838671	ZC2HC1C	0.7767285	CDK5	0.705987
FABP6	1.004217	METTL18	0.836372	NDC80	0.7749395	NDUFAF3	0.705917
HIST1H3G	1.0029625	ASPM	0.83573	DOLK	0.774007	FAM86C1	0.705856
DARS2	0.9981075	PEBP1	0.835273	ACADSB	0.773414	CAMKV	0.7043205
TIGAR	0.9933485	CYB561	0.835042	C12orf66	0.7729535	SPA17	0.703667
GABRB1	0.9921695	MGST2	0.834794	GSTA4	0.7719455	PLBD1	0.7035385
LPCAT1	0.9898065	ANAPC15	0.8332625	NXPH2	0.7708635	UMPS	0.7030695
HIST1H1D	0.9867975	RELL1	0.8310665	RORA	0.7702965	CTSF	0.7027555
C9orf64	0.984354	C6orf120	0.830514	COA5	0.7682175	NDE1	0.70091
CETN3	0.980721	UHRF1	0.8301445	MRPL40	0.7676895	AK1	0.7006635
						BCHE	0.700625

Supplementary Table 6. Genes downregulated by palbociclib related to Fig. 6c and d.

Common 89 genes			
HS3ST3A1	PALM3	SEPT4	TNFAIP8
KLHL14	CDT1	PIM1	PAQR5
PCSK1	RNASEH2B	CDCA7L	DLX5
DDB2	PMAIP1	OAS3	ABCB4
ZNF385B	BHLHE40	NEMP2	MXD3
ORC1	NHS	DDX60	KIF15
AURKB	GPC3	XKR5	METTL7A
MIR503HG	CCBE1	ARHGEF39	HJURP
BARD1	RP11-214O1.2	KIF2C	HHIP
IMPA2	BHLHE41	HIRIP3	CTNNAL1
FAM72D	SOX2	CDCA7	KIF20A
KANK4	SPC24	FOXN4	CEP152
E2F7	RDM1	CDC20	TRIM59
PSRC1	LBR	C8orf88	FAM72C
GPM6A	E2F8	EXOSC8	ANP32B
MEGF6	NID1	PRR11	C21orf58
CRNDE	CTB-193M12.5	HMGB2	PDGFC
C2orf48	KDELC2	H1F0	
NDE1	TGM2	ITGA4	
CD302	TEX19	PRIM1	
ANKRD18B	BORA	CDCA3	
KIAA1644	MBOAT1	RARRES2	
SERTAD4	SKP2	NQO1	
ERFE	FAM72B	TOP2A	

Supplementary Table 7. A 149 gene signature related to Fig. 7.

CCNB1	DDX28	WDR77
CCNE1	NOP56	CTPS1
FUS	RRM2	SNRNP25
CLCN4	FEN1	SPA17
UNG	NCAPD2	POP1
ARMC6	MRPL40	RPA3
KIF4A	FAM83D	DLGAP5
MCM10	CHAF1B	MKI67
KLRG1	MCM6	ZC3H4
HN1	POP5	CDKN2C
NDC80	PIGW	TUBB4B
CCNA2	ZNF326	POLA2
MAGI1	TRIM59	SPC24
PSRC1	UHRF1	E2F8
UCP2	COA3	HMGB3
KIF23	FGD6	NDUFAF3
PTCD2	CENPF	SFXN2
BRI3BP	SLC25A53	PLK1
LMNB1	SLC35B4	MRPL24
CCNB2	CDCA8	PIK3R3
MCM2	UTP20	MAP2K6
MCM4	TPX2	ALDH1B1
RPUSD2	ANP32E	LRRC34
CKAP2L	GINS1	KDM8
DTD2	KPNA2	PRMT6
CDCA2	BIRC5	FAM86C1
JMJD8	FABP5	MANEAL
GJC1	SUV39H2	CBLN1
CCNF	AURKA	RMI1
ALG1	AURKB	ASPM
GEMIN5	TIPIN	PNRC2
EEF2KMT	GRWD1	TMSB15A
LBR	RANBP1	ACAD9
DARS2	KCTD17	CDCA3
ESPL1	GEMIN4	ACAT2
FKBP7	C1orf131	HNRNPM
GTF2E1	LCMT2	SRSF1
TTF2	GTSE1	NOL6
SFPQ	KNOP1	SPC25
KIF11	PTTG1	BUB1B
KNSTRN	PFAS	POLR3H
KIFC1	CENPE	HMMR
TMPO	MRM1	FAM217B
MRPS7	HN1L	GMNN
ARHGEF39	HNRNPA2B1	KIF20A
RUVBL1	TACC3	CCDC121
WDR4	NAE1	FDPS
CENPA	ICMT	AMD1
H2AFX	SLC2A6	NUSAP1
UROS	CEP55	

Supplementary Table 8. The gene list differentially expressed in high-risk and low-risk neuroblastomas related to Fig. 7.

Gene symbol	Class
RPA3	High-risk
KIF20A	High-risk
SFXN2	High-risk
MCM6	High-risk
RPUSD2	High-risk
MKI67	High-risk
NUSAP1	High-risk
PTTG1	High-risk
DARS2	High-risk
CDCA2	High-risk
MCM2	High-risk
GMNN	High-risk
LMNB1	High-risk
UNG	High-risk
BUB1B	High-risk
POLA2	High-risk
HNRNPM	High-risk
SUV39H2	High-risk
WDR77	High-risk
FAM83D	High-risk
CCNE1	High-risk
KIF23	High-risk
MCM4	High-risk
MCM10	High-risk
GINS1	High-risk
TPX2	High-risk
SNRNP25	High-risk
TMPO	High-risk
CENPF	High-risk
NCAPD2	High-risk
CHAF1B	High-risk
TTF2	High-risk
DDX28	High-risk
AURKA	High-risk
DLGAP5	High-risk
ANP32E	High-risk
RRM2	High-risk
CENPA	High-risk
ARMC6	High-risk
CCNA2	High-risk
TUBB4B	High-risk
FEN1	High-risk
PLK1	High-risk
HN1	High-risk
TIPIN	High-risk
RANBP1	High-risk
CCNB1	High-risk
SRSF1	High-risk
MRPS7	High-risk
CENPE	High-risk
H2AFX	High-risk
KIFC1	High-risk
KPNA2	High-risk
KIF11	High-risk
SPC25	High-risk
TRIM59	High-risk
TACC3	High-risk
POP1	High-risk
GRWD1	High-risk
CDCA8	High-risk
SPC24	High-risk
TMSB15A	High-risk
NDC80	High-risk
E2F8	High-risk
GTSE1	High-risk
AURKB	High-risk
CBLN1	High-risk
HMGB3	High-risk
UHRF1	High-risk
BRI3BP	High-risk
ESPL1	High-risk
UTP20	High-risk
GJC1	High-risk
KNSTRN	High-risk
CKAP2L	High-risk
ASPM	High-risk
CEP55	High-risk
RUVBL1	High-risk
BIRC5	High-risk
HMMR	High-risk
PSRC1	High-risk
LBR	High-risk
KIF4A	High-risk
CCNB2	High-risk
DTD2	High-risk
ARHGEF39	Low risk
ICMT	Low risk
PNRC2	Low risk
POP5	Low risk
SFPQ	Low risk
SLC35B4	Low risk
POLR3H	Low risk
MANEAL	Low risk
CDKN2C	Low risk
CCDC121	Low risk
ALG1	Low risk
KDM8	Low risk
SLC2A6	Low risk
PIK3R3	Low risk
JMJD8	Low risk
KLRG1	Low risk
UCP2	Low risk
FAM217B	Low risk
FGD6	Low risk
MAGI1	Low risk
