# Supplementary material for: KDM6B promotes activation of the oncogenic CDK4/6-pRB-E2F pathway by maintaining enhancer activity in MYCN-amplified neuroblastoma
Source: Nat Commun. 2021 Dec 10;12:7204. doi: 10.1038/s41467-021-27502-2 (PMC8664842; doi:10.1038/s41467-021-27502-2)
Supplement: Supplementary file 3 — Description of Additional Supplementary Files [file 41467_2021_27502_MOESM3_ESM.pdf]

Description of Additional Supplementary Files

File Name: Supplementary Data 1
Description: The differential H3K27me3 peaks induced by KDM6B knockdown and GSK-J4 treatment.

File Name: Supplementary Data 2
Description: The differential H3K4me1 peaks induced by KDM6B knockdown and GSK-J4 treatment.
