# Supplementary material for: KDM6B promotes activation of the oncogenic CDK4/6-pRB-E2F pathway by maintaining enhancer activity in MYCN-amplified neuroblastoma
Source: Nat Commun. 2021 Dec 10;12:7204. doi: 10.1038/s41467-021-27502-2 (PMC8664842; doi:10.1038/s41467-021-27502-2)
Supplement: Supplementary file 6 — Reporting Summary [file 41467_2021_27502_MOESM6_ESM.pdf]

nature portfolio

Corresponding author(s): Jun Yang

Last updated by author(s): Nov 4, 2021

Reporting Summary

Nature Portfolio wishes to improve the reproducibility of the work that we publish. This form provides structure for consistency and transparency in reporting. For further information on Nature Portfolio policies, see our Editorial Policies and the Editorial Policy Checklist.

Statistics

For all statistical analyses, confirm that the following items are present in the figure legend, table legend, main text, or Methods section.

n/a	Confirmed	
☐	☒	The exact sample size (n) for each experimental group/condition, given as a discrete number and unit of measurement
☐	☒	A statement on whether measurements were taken from distinct samples or whether the same sample was measured repeatedly
☐	☒	The statistical test(s) used AND whether they are one- or two-sided<br>*Only common tests should be described solely by name; describe more complex techniques in the Methods section.*
☒	☐	A description of all covariates tested
☒	☐	A description of any assumptions or corrections, such as tests of normality and adjustment for multiple comparisons
☐	☒	A full description of the statistical parameters including central tendency (e.g. means) or other basic estimates (e.g. regression coefficient) AND variation (e.g. standard deviation) or associated estimates of uncertainty (e.g. confidence intervals)
☐	☒	For null hypothesis testing, the test statistic (e.g. F, t, r) with confidence intervals, effect sizes, degrees of freedom and P value noted<br>*Give P values as exact values whenever suitable.*
☒	☐	For Bayesian analysis, information on the choice of priors and Markov chain Monte Carlo settings
☒	☐	For hierarchical and complex designs, identification of the appropriate level for tests and full reporting of outcomes
☐	☒	Estimates of effect sizes (e.g. Cohen's d, Pearson's r), indicating how they were calculated

Our web collection on statistics for biologists contains articles on many of the points above.

Software and code

Policy information about availability of computer code

Data collection	RNA-seq data were collected by Paired-end sequencing was performed using the Illumina HiSeq 2500 with 100bp read length. Affymetrix microarray data were generated by Affymetrix Clariom S human array. ATAC-seq were collected by Paired-end 2X100bp sequencing with Illumina HiSeq 4000. Motifs were downloaded from TRANSFEC (v2018.3). Cut&Tag data were collected by the Paired-end sequencing with Illumina HiSeq2500 with 50bp read length.
Data analysis	Commercial and publicly available softwares were used to analyze data.<br>For RNA-seq data analysis, STAR 2.5.3a, HTSeq 0.6.1p, VOOM, LIMMA 3.26.9 were used.<br>For Affymetrix Clariom S human array data, GenePattern program versioin 3.9.11 was used.<br>For ATAC-seq data analysis, cutadapt version 1.9, BWA version 0.7.12-r1039, Picard 2.6.0-SNAPSHOT, samtools version 1.2, MACS2 version 2.1.1, IGV version 2.4.13, Bedtools version 2.24.0(mergeBed), R version 3.23, Voom function from edgeR 3.12.1, limma 3.26.9, MEME suite v4.11.3, Fisher exact test using stats module from Scipy 1.0.0, were used.<br>For Cut&Tag data analysis, SPP version 1.1, Picard 1.6.5 , BWA version 0.7.12, MACS versoin 2.2.7.1, SICER version 1.1, bedtools version 2.25.0,samtools version 1.3.1, BigWigtoBedGraph, bigWigMerge, bedGraphTobigWig, IGV version 2.4.13, DeepTools version 2.3, MergeBed, annotatePeaks.pl, edgeR 3.16.5, Homer software version 4.8.3 were used.<br>GSEA 4.1.0 program was used for analyzing gene pathway enrichment.<br>Other programs including Microsoft Excel version 16.54, Adobe Phostoshop version 2021, Adobe Illustrator version 2021, Prism GraphPad 9 were used to present and analyze data.

For manuscripts utilizing custom algorithms or software that are central to the research but not yet described in published literature, software must be made available to editors and reviewers. We strongly encourage code deposition in a community repository (e.g. GitHub). See the Nature Portfolio guidelines for submitting code & software for further information.

Data

Policy information about availability of data

All manuscripts must include a data availability statement. This statement should provide the following information, where applicable:

- Accession codes, unique identifiers, or web links for publicly available datasets
- A description of any restrictions on data availability
- For clinical datasets or third party data, please ensure that the statement adheres to our policy

The RNA-seq, ATAC-seq, Cut&Tag raw data have been deposited to the Gene Expression Omnibus-GEO (NCBI) as superseries, under accession number GSE149539 (https://www.ncbi.nlm.nih.gov/gds/?term=GSE149539[Accession]). The Affymetrix microarray data have been deposited to GEO under accession number GSE150045 (https://www.ncbi.nlm.nih.gov/geo/query/acc.cgi?acc=GSE150045). Databases/datasets used in the study were the following: Library of Integrated Network-based Cellular Signatures (LINCS)(https://maayanlab.cloud/Enrichr/), The Cancer Therapeutics Response Portal (CTRP) (https://portals.broadinstitute.org/ctrp.v2.1/), R2: Genomics Analysis and Visualization Platform (https://hgserver1.amc.nl/cgi-bin/r2/main.cgi), Cancer Cell Line Encyclopedia-CCLE(https://sites.broadinstitute.org/ccle/), St Jude Pecan Portal (https://pecan.stjude.cloud/; https://pecan.stjude.cloud/proteinpaint/study/mycn_nbl_2018), CellMinerCDB (https://discover.nci.nih.gov/rsconnect/cellminercdb/). TRANSFEC (https://genexplain.com/transfac/). The authors declare that other data supporting the findings of this study are provided in the Supplementary information/Data/Source Data files. Uncropped western blot images are present in Source Data file. Source data files are provided with this paper.

Field-specific reporting

Please select the one below that is the best fit for your research. If you are not sure, read the appropriate sections before making your selection.

☒ Life sciences ☐ Behavioural & social sciences ☐ Ecological, evolutionary & environmental sciences

For a reference copy of the document with all sections, see nature.com/documents/nr-reporting-summary-flat.pdf

Life sciences study design

All studies must disclose on these points even when the disclosure is negative.

Sample size	Experiments were designed to have enough sample sizes to obtain reliable results. Neural crest tissue sample size n=5, Versteeg tumor sample size n=88, Delattre tumor sample size n=64, Hiyama tumor sample size n=51, , Lastowska tumor sample size n=30, St Jude dataset tumor sample size =160, TARGET dataset tumor sample size n=161, SEQC dataset tumor sample size n=498. For in vitro experiments, at least three independent replicates and a minimum of two biological replicates were used for each experiment to ensure the reproducibility and to perform statistical analysis.
Data exclusions	No data exclusion
Replication	Data was obtained from three technical replicates and in at least two biological replicates. Independent biological replicates are shown in all figures.
Randomization	Randomization was not relevant. All cell lines or biological samples were analyzed or treated in the same manner.
Blinding	Experiments were not blinded in order to allow the investigators to have correct identification of samples and to ensure the correct data collection. In other hand, blinding strategy was applied to computational biologists who performed bioinformatic analyses.

Reporting for specific materials, systems and methods

We require information from authors about some types of materials, experimental systems and methods used in many studies. Here, indicate whether each material, system or method listed is relevant to your study. If you are not sure if a list item applies to your research, read the appropriate section before selecting a response.

Materials & experimental systems

n/a	Involved in the study	
☐	☒	Antibodies
☐	☒	Eukaryotic cell lines
☒	☐	Palaeontology and archaeology
☒	☐	Animals and other organisms
☒	☐	Human research participants
☒	☐	Clinical data
☒	☐	Dual use research of concern

Methods

n/a	Involved in the study	
☐	☒	ChIP-seq
☒	☐	Flow cytometry
☒	☐	MRI-based neuroimaging

Antibodies

Antibodies used

H3K27me3 Cell Signaling Technology Cat# 9733, RRID:AB_2616029, 1:1000 for western blot, 1:50 for CUT&Tag
H3K4me1 Abcam Cat# ab8895, RRID:AB_306847, 1:1000 for western blot, 1:50 for CUT&Tag
H3K4me3 RevMAb Biosciences Cat# 31-1226-00, RRID:AB_2783580, 1:2000
H3K27ac Active Motif Cat# 39133, RRID:AB_2561016, 1:1000 for western blot
Total H3 Cell Signaling Technology Cat#4499, RRID: AB_10544537, 1:5000 for western blot
CDK4 Cell Signaling Technology Cat# 12790, RRID:AB_2631166, 1:1000 for western blot
CDK6 Cell Signaling Technology Cat# 13331, RRID:AB_2721897, 1:1000 for western blot
β-ACTIN Sigma-Aldrich Cat# A1978, RRID:AB_476692, 1:5000 for western blot
KDM6B Abcam Cat# 154126, RRID:AB_2722742, 1:1000 for western blot
KDM6B, ABclonal, Inc., Cat#A12763, RRID:AB_2759609, 1:1000 for western blot
RB1 (4H1) Cell Signaling Technology Cat# 9309, RRID:AB_823629, 1:2000 for western blot
N-MYC Cell Signaling Technology Cat# 9405, RRID:AB_10692664, 1:1000 for western blot
N-MYC Santa Cruz Biotechnology Cat# sc-53993, RRID:AB_831602, 1:200 for western blot
C-MYC Cell Signaling Technology Cat# 13987, RRID:AB_2631168, 1:1000 for western blot
C-Myc, Cell Signaling Technology, Cat# 5605, RRID:AB_1903938, 1:1000 for western blot
GAPDH Cell Signaling Technology Cat# 5174, RRID:AB_10622025, 1:1000 for western blot
H3K9me3, Active Motif, Cat# 39161, RRID:AB_2532132, 1:1000 for western blot
H3K36me3, Abcam, Cat# ab9050, RRID:AB_306966, 1:1000 for western blot
Secondary horseradish peroxidase (HRP)-conjugated goat anti-mouse, Thermo Fischer Scientific Cat#31430, RRID:AB_228307,1:5000 for western blot
Secondary horseradish peroxidase (HRP)-conjugated goat anti-rabbit, Thermo Fischer Scientific Cat#31460, RRID:AB_228341, 1:5000 for western blot

Validation

All antibodies were purchased from commercial vendors and were validated by manufactures, other studies and/or in this study. We also provided associated datasheets link as below. Please note some antibodies may have been discontinued.

H3K27me3 (Cell Signaling Technology, Cat# 9733) website validation: 556 citations. Western blot analysis successfully stained in various cell lines lysis.
https://www.cellsignal.com/products/primary-antibodies/tri-methyl-histone-h3-lys27-c36b11-rabbit-mab/9733
CUT&Tag validation: Kaya-Okur, H. S., Janssens, D. H., Henikoff, J. G., Ahmad, K. & Henikoff, S. Efficient low-cost chromatin profiling with CUT&Tag. Nat Protoc 15, 3264-3283, doi:10.1038/s41596-020-0373-x (2020)

H3K4me1 (Abcam, Cat#ab8895) website validation: 803 citations. Western blot analysis successfully stained in various cell lines lysis.
https://www.abcam.com/histone-h3-tri-methyl-k36-antibody-chip-grade-ab9050.html
CUT&Tag validation: Kaya-Okur, H. S., Janssens, D. H., Henikoff, J. G., Ahmad, K. & Henikoff, S. Efficient low-cost chromatin profiling with CUT&Tag. Nat Protoc 15, 3264-3283, doi:10.1038/s41596-020-0373-x (2020)

H3K4me3 (RevMAb Biosciences, Cat# 31-1226-00):
https://www.revmab.com/index.php/product/anti-trimethyl-histone-h3-lys4-rabbit-monoclonal-antibody-clone-rm340-h3k4me3-histone-h3-trimethyl-k4/

H3K27ac (Active Motif, Cat# 39133) website validation: 65 citations. https://www.activemotif.com/catalog/details/39133

Total H3 (Cell Signaling Technology, Cat#4499):
https://www.cellsignal.com/products/primary-antibodies/histone-h3-d1h2-xp-rabbit-mab/4499

CDK4 (Cell Signaling Technology, Cat# 12790):
https://www.cellsignal.com/products/primary-antibodies/cdk4-d9g3e-rabbit-mab/12790

CDK6 (Cell Signaling Technology, Cat# 13331):
https://www.cellsignal.com/products/primary-antibodies/cdk6-d4s8s-rabbit-mab/13331

β-ACTIN (Sigma-Aldrich, Cat# A1978):
https://www.sigmaaldrich.com/US/en/product/sigma/a1978

KDM6B (Abcam Cat# 154126) validated in this study by siRNA knockdown and overexpression.
This antibody is discontinued.
https://www.abcam.com/kdm6b--jmjd3-antibody-c-terminal-ab154126.html

KDM6B (KDM6B, ABclonal, Inc., Cat#A12763) is validated in this study by siRNA knockdown and overexpression, and in company website.
https://abclonal.com/catalog-proteins/Kdm6bRabbitpAb/A12763

RB1 (4H1) (Cell Signaling Technology, Cat# 9309):
https://www.cellsignal.com/products/primary-antibodies/rb-4h1-mouse-mab/9309

N-MYC (Cell Signaling Technology, Cat# 9405):
https://www.cellsignal.com/products/primary-antibodies/n-myc-antibody/9405

N-MYC (Santa Cruz Biotechnology, Cat# sc-53993), website validation: 156 citations.

https://www.scbt.com/p/n-myc-antibody-b8-4-b

C-MYC (Cell Signaling Technology, Cat# 13987):
https://www.cellsignal.com/products/primary-antibodies/c-myc-n-myc-d3n8f-rabbit-mab/13987

C-Myc (Cell Signaling Technology, Cat# 5605):
https://www.cellsignal.com/products/primary-antibodies/c-myc-d84c12-rabbit-mab/5605

GAPDH (Cell Signaling Technology, Cat# 5174):
https://www.cellsignal.com/products/primary-antibodies/gapdh-d16h11-xp-rabbit-mab/5174

H3K9me3 (Active Motif, Cat# 39161):
https://www.activemotif.com/catalog/details/39161

H3K36me3 (Abcam, Cat# ab9050), website validation: 803 citations.
https://www.abcam.com/histone-h3-tri-methyl-k36-antibody-chip-grade-ab9050.html

Secondary horseradish peroxidase (HRP)-conjugated goat anti-mouse (Thermo Fischer Scientific Cat#31430):
https://www.thermofisher.com/antibody/product/Goat-anti-Mouse-IgG-H-L-Secondary-Antibody-Polyclonal/31430

Secondary horseradish peroxidase (HRP)-conjugated goat anti-rabbit (Thermo Fischer Scientific Cat#31460):
https://www.thermofisher.com/antibody/product/Goat-anti-Rabbit-IgG-H-L-Secondary-Antibody-Polyclonal/31460

Eukaryotic cell lines

Policy information about cell lines

Cell line source(s)	Human: BE2C ATCC CRL-2268 RRID:CVCL_0529<br>Human: SIMA DSMZ ACC 164 RRID:CVCL_1695<br>Human: U2OS ATCC HTB-96 RRID:CVCL_0042<br>Human: 293T ATCC CRL-3216 RRID:CVCL_0063<br>Human: SK-N-DZ ATCC CRL-2149 RRID:CVCL_1701<br>Human: SK-N-AS ATCC CRL-2137 RRID:CVCL_1700<br>Human: SK-N-SH ATCC HTB-11 RRID:CVCL_0531<br>Human: SK-N-FI ATCC CRL-2142 RRID:CVCL_1702<br>Human: KELLY ECACC 92110411 RRID:CVCL_2092<br>Human: SKNBE2 COG RRID:CVCL_0528<br>Human: IMR32 ATCC CL-127 RRID:CVCL_0346<br>Human: CHLA15 COG RRID:CVCL_6594<br>Human: CHLA20 COG RRID:CVCL_6602<br>Human: NB-1691 Peter Houghton RRID:CVCL_5628<br>Human: NB-1643 Peter Houghton RRID:CVCL_5627<br>Human: HS68 ATCC CRL-1635 RRID:CVCL_0839<br>Human: HCT116 ATCC, RRID: CCL_247<br>Human: HCT116,p53-/- Dr. Bert Vogelstein lab<br>Human: A549 ATCC CCL-185 RRID:CVCL_0023<br>Human: BE2C-CDK4-OE This paper<br>Human: BE2C-CDK6-OE This paper<br>Human: BE2C-RB1-KO This paper
Authentication	Cell lines were authenticated by short tandem repeat (STR) using Promega PowerPlex 16 HS System once per month.
Mycoplasma contamination	PCR-based method was used for detection of Mycoplasma with LookOut Mycoplasma PCR Detection Kit (Sigma) and JumpStart Taq DNA Polymerase (Sigma) once per month to ensure cells were mycoplasma negative.
Commonly misidentified lines<br>(See ICLAC register)	No commonly misidentified cell lines were used in this study.

ChIP-seq

Data deposition

☒ Confirm that both raw and final processed data have been deposited in a public database such as GEO.

☐ Confirm that you have deposited or provided access to graph files (e.g. BED files) for the called peaks.

Data access links<br>*May remain private before publication.*	https://www.ncbi.nlm.nih.gov/gds/?term=GSE149539[Accession]
Files in database submission	2190698_BE2C-siCtrl-H3K4me1-1.bam

Files in database submission

2190699_BE2C-siCtrl-H3K4me1-2.bam
2190700_BE2C-siCtrl-H3K27me3-1.bam
2190701_BE2C-siCtrl-H3K27me3-2.bam
2190702_BE2C-si6B33-H3K4me1-1.bam
2190703_BE2C-si6B33-H3K4me1-2.bam
2190704_BE2C-si6B33-H3K27me3-1.bam
2190705_BE2C-si6B33-H3K27me3-2.bam
2216739_BE2C-DMSO-H3K4me1-1.bam
2216740_BE2C-DMSO-H3K4me1-2.bam
2216741_BE2C-DMSO-H3K27me3-1.bam
2216742_BE2C-DMSO-H3K27me3-2.bam
2216743_BE2C-GSKJ4-H3K4me1-1.bam
2216744_BE2C-GSKJ4-H3K4me1-2.bam
2216745_BE2C--GSKJ4-H3K27me3-1.bam
2216746_BE2C--GSKJ4-H3K27me3-2.bam

Genome browser session (e.g. UCSC)

n/a

Methodology

Replicates

Two replicates per treatment

Sequencing depth

id Total UniqelyMapped read length single or paired-end
2190698_BE2C-siCtrl-H3K4me1-1 95,348,438 18,740,560 36 paired-end
2190699_BE2C-siCtrl-H3K4me1-2 84,403,362 17,829,001 36 paired-end
2190700_BE2C-siCtrl-H3K27me3-1 59,796,588 8,098,406 36 paired-end
2190701_BE2C-siCtrl-H3K27me3-2 67,670,148 8,228,918 36 paired-end
2190702_BE2C-si6B33-H3K4me1-1 113,509,424 22,738,862 36 paired-end
2190703_BE2C-si6B33-H3K4me1-2 107,030,832 22,455,279 36 paired-end
2190704_BE2C-si6B33-H3K27me3-1 70,549,144 10,919,079 36 paired-end
2190705_BE2C-si6B33-H3K27me3-2 75,296,930 10,772,371 36 paired-end
2216739_BE2C-DMSO-H3K4me1-1 84,236,406 12,828,690 36 paired-end
2216740_BE2C-DMSO-H3K4me1-2 66,989,164 10,885,805 36 paired-end
2216741_BE2C-DMSO-H3K27me3-1 54,873,664 7,257,169 36 paired-end
2216742_BE2C-DMSO-H3K27me3-2 47,211,056 8,338,370 36 paired-end
2216743_BE2C-GSKJ4-H3K4me1-1 81,382,546 12,697,583 36 paired-end
2216744_BE2C-GSKJ4-H3K4me1-2 73,548,156 13,008,656 36 paired-end
2216745_BE2C--GSKJ4-H3K27me3-1 50,040,762 12,599,936 36 paired-end
2216746_BE2C--GSKJ4-H3K27me3-2 38,220,538 8,975,504 36 paired-end

Antibodies

H3K27me3 Cell Signaling Technology Cat# 9733, RRID:AB_2616029
H3K4me1 Abcam Cat# ab8895, RRID:AB_306847

Peak calling parameters

Information in Supplementary Data 1 and 2

Data quality

Data quality was assessed by SPP (version 1.11).

Software

BWA (version 0.7.12)
Picard (version 1.65)
amtools (version 1.3.1)
R (version 3.5.1)
IGV (version 2.3.82)
genomeCoverageBed (bedtools 2.25.0)
MACS2 (version 2.1.1)
SICER (version 1.1)
HOMER suite (v4.8.3)
